# RHBDL2 drives lipid metabolic reprogramming in osteosarcoma via USP3-mediated deubiquitination of PPT1

**DOI:** 10.1038/s41419-026-08788-w

**Published:** 2026-04-24

**Authors:** Li Fan, Cheng Tao, Xiangwei Zeng, Jianpeng Liu, Rubo Cao, Kewei Zhu

**Affiliations:** 1 Cancer Center, Union Hospital, Tongji Medical College, Huazhong University of Science and Technology, Wuhan, China; 2 Department of Orthopedics, The Second Xiangya Hospital of Central South University, Changsha, China; 3 College of Medicine and Health Science, Wuhan Polytechnic University, Wuhan, Hubei PR China; 4 Personalised Oncology Division, The Walter and Eliza Hall Institute of Medical Research, Parkville, VIC Australia

**Keywords:** Cancer metabolism, Ubiquitylation

## Abstract

Osteosarcoma (OS) is characterized by high malignancy and profound metabolic reprogramming, yet the upstream regulators of its lipid metabolic adaptations remain largely elusive. Here, we report that RHBDL2 is significantly overexpressed in OS tissues, correlating with advanced clinical stage and poor patient prognosis. Mechanistically, multi-omics and structural analyses reveal that RHBDL2 functions as a non-proteolytic scaffold to stabilize the deubiquitinase USP3. This interaction is mediated by a compact hydrophobic core anchored by the Val245 residue of RHBDL2 and occurs independently of its protease activity. Stabilized USP3 subsequently prevents the proteasomal degradation of Palmitoyl-Protein Thioesterase 1 (PPT1) through deubiquitination. We further identify PPT1 as a metabolic rheostat that fuels OS malignancy by orchestrating FASN-dependent de novo lipogenesis, a requirement that can be partially bypassed by exogenous lipid supplementation. This RHBDL2-USP3-PPT1 axis promotes OS cell proliferation, migration, and epithelial-mesenchymal transition while suppressing apoptosis. Pharmacological screening identified Epigallocatechin gallate (EGCG) as a potent inhibitor that competitively disrupts the RHBDL2-USP3 interaction interface, thereby suppressing the downstream lipogenic program and inhibiting tumor growth and bone destruction in vivo. Collectively, our findings delineate a novel signaling cascade linking post-translational protein stabilization to metabolic adaptation, highlighting the RHBDL2-USP3 structural interface as a promising therapeutic vulnerability in osteosarcoma.

## Introduction

Osteosarcoma (OS), a highly malignant primary bone tumor predominantly affecting children and adolescents, poses significant clinical challenges due to its rapid progression, high metastatic propensity, and poor prognosis [1, 2]. The tumor’s molecular heterogeneity drives distinct subtypes, with lipid metabolic dysregulation emerging as a core feature of its malignant progression [1, 3]. Studies demonstrate that osteosarcoma cells reprogram lipid metabolism to fuel energy demands and sustain proliferation. For instance, exosome-mediated transfer of the XIST lncRNA upregulates ACLY expression, promoting lipid deposition and activating the β-catenin signaling pathway to drive tumor growth and metastasis [4]. Additionally, lipid metabolism-associated lncRNAs such as RPARP-AS1 modulate the expression of key enzymes like FASN, critically influencing tumor cell proliferation and invasive capabilities [5]. However, the upstream regulators orchestrating these metabolic adaptations remain poorly understood.

RHBDL2 (Rhomboid-like protein 2), a transmembrane protein belonging to the rhomboid protease family, regulates substrate cleavage and signaling pathways. Emerging studies highlight its pro-tumorigenic roles in multiple cancers. In pancreatic cancer, RHBDL2 promotes tumor proliferation and metastasis by activating the Notch signaling pathway. Specifically, it cleaves Notch1 to release its intracellular domain (N1ICD) and stabilizes N1ICD activity through synergistic interaction with the deubiquitinating enzyme OTUD7B, which inhibits N1ICD ubiquitination and degradation [6]. In clear cell renal cell carcinoma (ccRCC), RHBDL2 drives malignancy by suppressing cuproptosis (a copper-dependent cell death pathway) and activating the Wnt/β-catenin signaling axis [7]. Its dysregulation may also foster an immunosuppressive tumor microenvironment (TME) by enhancing regulatory T cell (Treg) infiltration, thereby facilitating immune evasion [7]. In gastric cancer, RHBDL2 facilitates the epithelial-mesenchymal transition (EMT) through activation of the PI3K/AKT signaling pathway. Notably, when cyclin B2 expression is suppressed, the overexpression of RHBDL2 is capable of restoring the EMT process, thereby indicating its critical role in gastric cancer metastasis [8]. Together, RHBDL2 drives tumorigenesis through multi-pathway crosstalk, but its specific roles in osteosarcoma require experimental validation.

Palmitoyl-Protein Thioesterase 1 (PPT1) is a lysosomal enzyme that catalyzes the depalmitoylation of S-palmitoylated proteins, thereby playing a critical role in the dynamic regulation of protein post-translational modifications [9]. This enzyme has been implicated in various pathological conditions, including neurodegenerative diseases, cancer, and metabolic disorders. In recent years, its involvement in tumorigenesis, disease progression, and potential therapeutic strategies has garnered significant attention. PPT1 modulates the survival and proliferation of tumor cells by influencing the metabolism, localization, and functionality of macromolecular proteins. For instance, in hepatocellular carcinoma (HCC), PPT1 enhances the metabolic fitness of tumor cells by regulating the degradation of palmitoylated proteins and maintaining lysosomal function [10]. As a key regulator of the autophagy-lysosomal pathway, PPT1 inhibition disrupts autophagy, induces tumor cell apoptosis, and augments the efficacy of anti-PD-1 immunotherapy [11]. In addition, the PPT1 inhibitor Ezurpimtrostat, in combination with PD-1 blockade, enhances the regeneration of CD8^+^ T cells in HCC, thereby improving T cell-mediated immunotherapy for HCC [12]. Research has demonstrated that PPT1 is predominantly expressed in macrophages within HCC and contributes to the immunosuppressive transformation of macrophages and the tumor microenvironment. The infiltration of PPT1-positive macrophages correlates with an unfavorable prognosis in HCC patients, indicating that PPT1 may facilitate tumor progression by modulating the immune microenvironment [13]. Additionally, PPT1 regulates angiogenesis by controlling the palmitoylation level of glutathione peroxidase 1 (Gpx1). Inhibition of PPT1 effectively suppresses pathological angiogenesis by enhancing Gpx1 palmitoylation [14]. Current studies on the role of PPT1 in osteosarcoma are limited. Exploring its mechanisms and clinical relevance could deepen insights into tumor pathogenesis and advance therapeutic strategies to improve patient outcomes.

Here, we uncover a RHBDL2-USP3-PPT1 axis that drives lipid metabolic reprogramming and OS progression. We demonstrate that RHBDL2 stabilizes PPT1 by enhancing USP3 expression to remove ubiquitin chains, thereby enhancing lipid synthesis and storage. This axis promotes OS cell proliferation, migration, and tumor growth while suppressing apoptosis. Our findings reveal a previously unrecognized mechanism linking proteolytic regulation to metabolic adaptation in OS, offering a promising therapeutic target for this recalcitrant malignancy.

## Results

### High expression of RHBDL2 correlates with poor prognosis and promotes the proliferation and migration of osteosarcoma cells

**Fig. 1:**
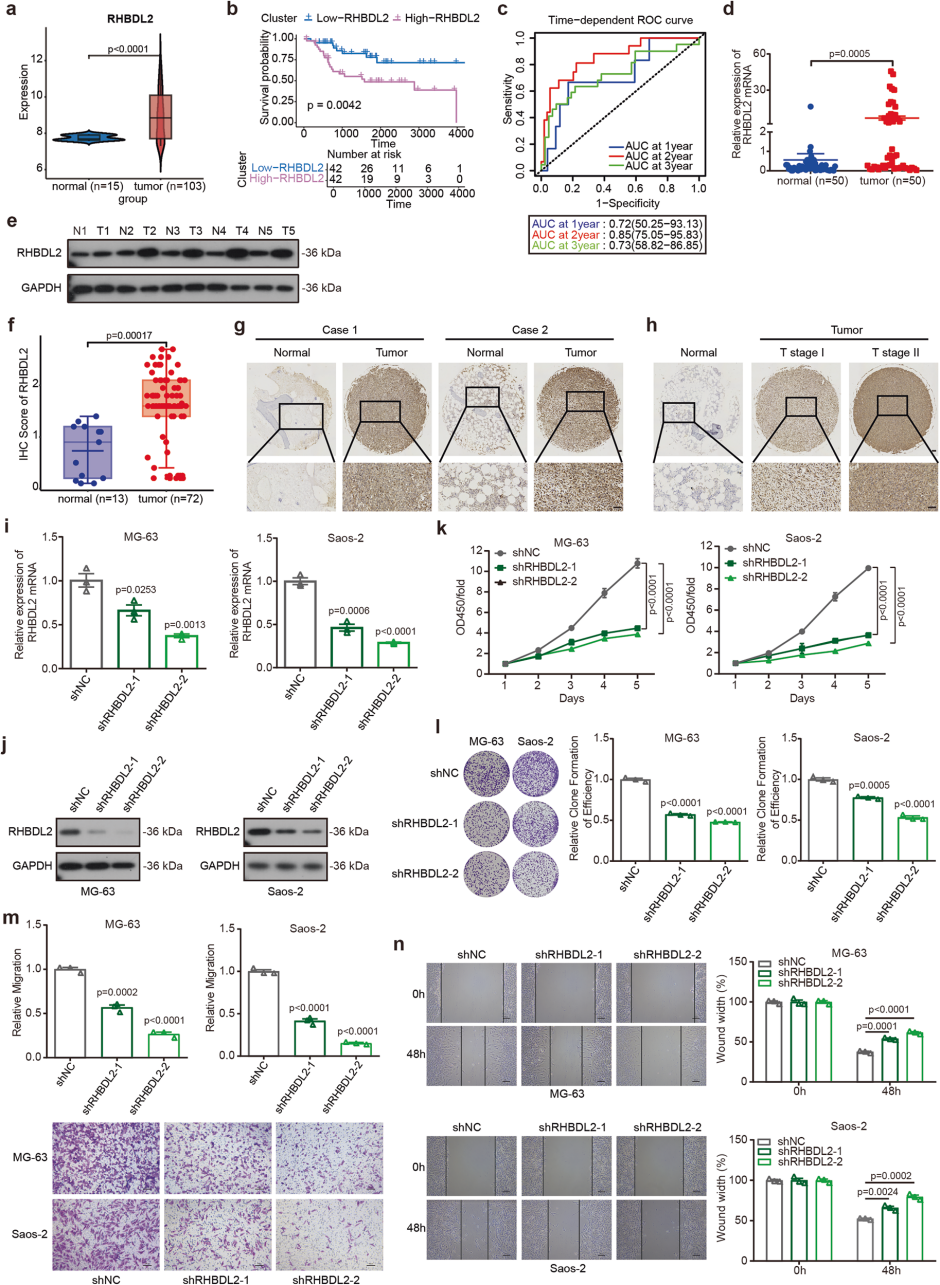
RHBDL2 is upregulated in osteosarcoma and promotes its malignant progression. **a** Relative mRNA expression of RHBDL2 in normal versus tumor tissues according to the GSE42352 database. **b** Kaplan–Meier survival analysis of OS patients stratified by RHBDL2 expression levels (Low vs. High). **c** ROC curve analysis of RHBDL2 expression for predicting 1-, 2-, and 3-year survival (AUCs: 0.72, 0.85, and 0.73, respectively). **d** Relative mRNA expression of RHBDL2 in 50 pairs of normal tissues and OS tissues. **e** Validation of RHBDL2 protein expression in paired tumor (T) and adjacent normal (N) tissues from 5 representative cases by Western blot, with GAPDH as a loading control. **f** IHC scores for RHBDL2 in normal and OS tissues were obtained from tissue microarrays LN020Bn01 and L072Bn01 respectively. Immunohistochemical evaluation of RHBDL2 protein expression in normal tissue, OS tissues (**g**), and across ascending T stages of OS (**h**). Scale bar 100 μm (the upper row) or 50 μm (the lower row). **i** Relative mRNA expression of RHBDL2 in MG-63 and Saos-2 cells transfected with shRHBDL2-1, shRHBDL2-2, or non-targeting control shNC. Data shown as mean ± SD (*n* = 3 biologically independent experiments); unpaired *t*-test for significance. **j** Western blot confirming RHBDL2 protein knockdown in both cell lines, with GAPDH as a loading control. **k** CCK-8 assay demonstrating the effect of RHBDL2 knockdown on the proliferation of OS cells over 5 days. Data shown as mean ± SD (*n* = 3 biologically independent experiments); unpaired *t*-test for significance. **l** Colony formation assay showing the impact of RHBDL2 suppression on the clonogenic ability of OS cells. Representative images of colonies stained with crystal violet are shown. Data shown as mean ± SD (n = 3 biologically independent experiments); unpaired *t*-test for significance. **m** Transwell migration assay indicating the role of RHBDL2 in the migratory potential of OS cells. Representative images of stained cells are shown. Scale bar 100 μm. Data shown as mean ± SD (n = 3 biologically independent experiments); unpaired *t*-test for significance. **n** Wound healing assay illustrating the effect of RHBDL2 knockdown on cell migration in OS cells. Representative images of wound closure at 0 h and 48 h post-scratch are shown. Scale bar 100 μm. Data shown as mean ± SD (*n* = 3 biologically independent experiments); unpaired *t*-test for significance.

To evaluate the clinical significance and prognostic potential of RHBDL2 in osteosarcoma, we analyzed its expression levels in tumor and normal tissues. RHBDL2 was significantly overexpressed in OS tumor tissues compared to normal tissues, as evidenced by data from the GSE42352 database. (Fig. 1a). This overexpression was associated with poor prognosis in OS patients. Kaplan-Meier survival analysis indicated that high RHBDL2 expression correlated with lower survival probability (Fig. 1b). Time-dependent ROC curve analysis demonstrated that RHBDL2 expression levels have good predictive value for medium-term (2-year) survival (AUC = 0.85). The predictive value for short-term (1-year) and long-term (3-year) survival remained moderate, with AUC values of 0.72 and 0.73, respectively (Fig. 1c). In our validation experiments, both mRNA and protein levels of RHBDL2 were found to be significantly higher in tumor tissues than in adjacent normal tissues, as assessed by quantitative real-time PCR (Fig. 1d) and Western blot (Fig. 1e). Furthermore, IHC assay also showed that the RHBDL2 expression was dramatically upregulated in OS tissues, especially in patients with advanced T stage (Fig. 1f–h). Clinicopathological analysis showed that elevated RHBDL2 expression was closely associated with advanced T stage in OS patients (*p* = 0.017), though no significant correlation was found with age, sex, lymph node metastasis, or TNM stage (Table S1). In summary, RHBDL2 is overexpressed in osteosarcoma and serves as a potential prognostic biomarker for patients with this condition.

To clarify the role of RHBDL2 in the oncogenic phenotype of OS cells, we conducted a series of in vitro functional assays. First, Western blot and RT-qPCR confirmed the successful knockdown of PPT1 (Fig. 1i, j). Then, the effect of RHBDL2 knockdown on cell proliferation was assessed using the CCK-8 assay over five days. As a result, in both MG-63 and Saos-2 cells, RHBDL2 suppression significantly inhibited proliferation in a time-dependent manner (Fig. 1k). Furthermore, the colony formation assay was performed to evaluate the impact of RHBDL2 knockdown on the clonogenic ability of osteosarcoma cells. Both shRHBDL2-1 and shRHBDL2-2 transfections led to a significant reduction in the number and size of colonies formed by MG-63 and Saos-2 cells compared to the shNC group (Fig. 1l). Consequently, the relative clone formation efficiency was markedly lower in RHBDL2-suppressed cells, indicating impaired proliferative capacity. Additionally, RHBDL2 suppression also affected the migratory potential of osteosarcoma cells. The Transwell migration assay showed a significant decrease in the number of migrated cells in the shRHBDL2 groups compared to the shNC control (Fig. 1m). Similarly, the wound healing assay corroborated these findings, showing a significant reduction in wound closure rates in RHBDL2-knockdown cells compared to controls (Fig. 1n). Overall, the data collectively underscore RHBDL2 as a critical driver of tumorigenic behaviors in osteosarcoma.

### RHBDL2 modulates lipid metabolism in OS cells through PPT1

**Fig. 2:**
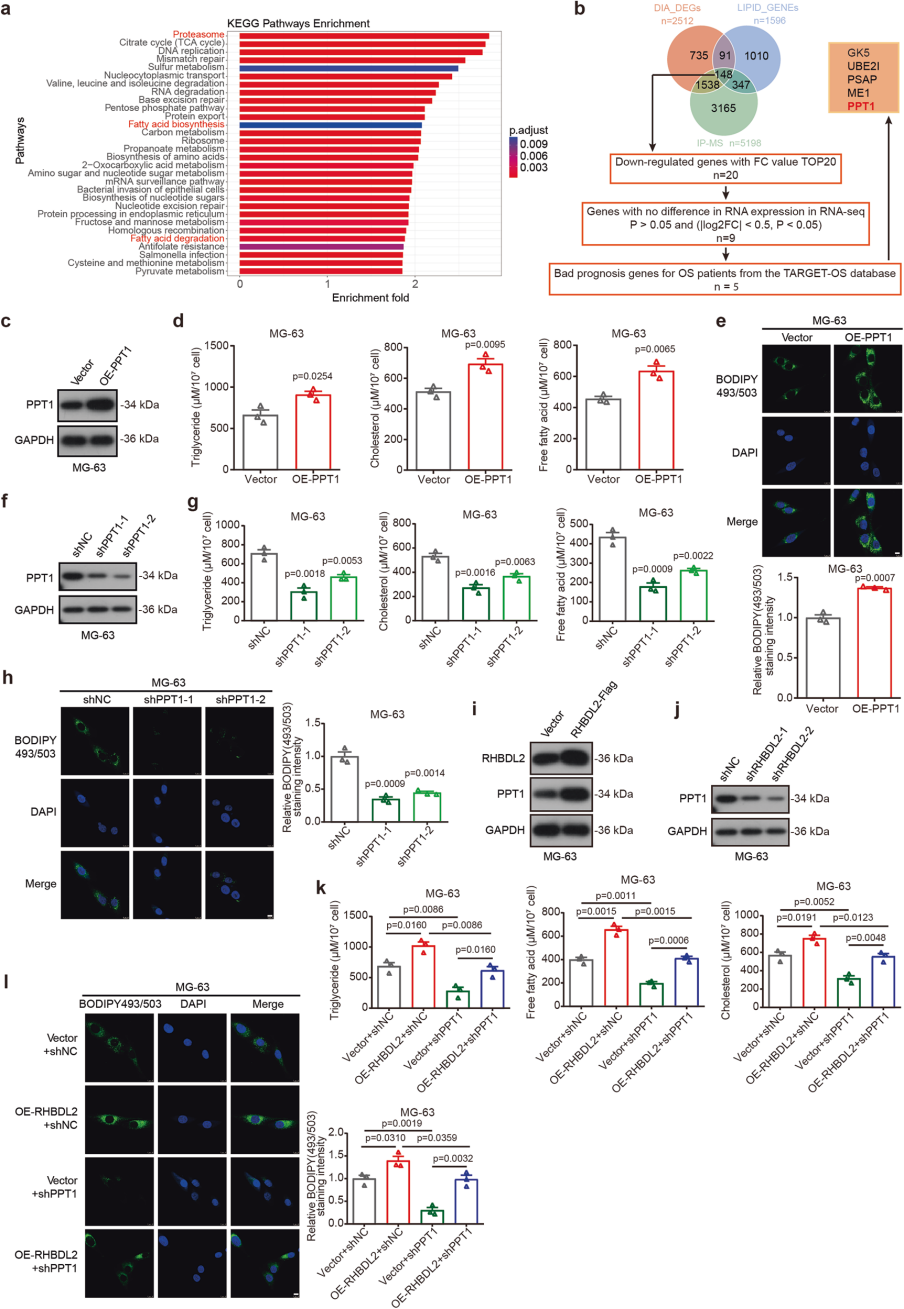
RHBDL2 regulates OS cell lipid metabolism via PPT1. **a** Enriched signaling pathways were identified by KEGG with proteins in IP-MS data. **b** Schematic diagram of the screening for the lipid metabolic genes that correlate with poor prognosis in OS. **c** Western blot of PPT1 protein levels in MG-63 cells transfected with vector or OE-PPT1. **d** Triglyceride, cholesterol, and free fatty acid levels in OE-PPT1 cells. Data shown as mean ± SD (*n* = 3 biologically independent experiments); unpaired *t*-test for significance. **e** Lipid droplet accumulation (BODIPY493/503 staining, DAPI nuclei) and quantification in OE-PPT1 cells. Scale bar 10 μm. Data shown as mean ± SD (*n* = 3 biologically independent experiments); unpaired *t*-test for significance. **f** Western blot analysis showing the protein expression levels of PPT1 in MG-63 cells transfected with shNC, shPPT1-1, or shPPT1-2. **g** Triglyceride, cholesterol, and free fatty acid levels in PPT1-knockdown cells. Data shown as mean ± SD (*n* = 3 biologically independent experiments); unpaired *t*-test for significance. **h** Lipid droplet imaging and quantification in PPT1-knockdown cells. Scale bar 10 μm. Data shown as mean ± SD (*n* = 3 biologically independent experiments); unpaired *t*-test for significance. **i** Western blot analysis showing the expression of RHBDL2 and PPT1 in MG-63 cells transfected with vector or RHBDL2-Flag. **j** Western blot analysis showing the expression of PPT1 in MG-63 cells transfected with shNC, shRHBDL2-1, or shRHBDL2-2. **k** Quantification of triglyceride, cholesterol, and free fatty acid levels in MG-63 cells co-transfected with vector or OE-RHBDL2 and shNC or shPPT1. Data shown as mean ± SD (*n* = 3 biologically independent experiments); unpaired *t*-test for significance. **l** Lipid droplet analysis in MG-63 cells co-transfected with vector or OE-RHBDL2 and shNC or shPPT1. Scale bar 10 μm. Data shown as mean ± SD (*n* = 3 biologically independent experiments); unpaired *t*-test for significance.

To elucidate the impact of RHBDL2 on the biological functions of osteosarcoma, we initially conducted KEGG pathway enrichment analyses from IP-MS data. The results revealed significant alterations in lipid metabolism-related pathways, including fatty acid degradation and biosynthesis (Fig. 2a; Supplementary Fig. 1a). To screen and identify lipid metabolism-related genes regulated by RHBDL2 in osteosarcoma, we performed an integrated analysis of multi-omics datasets from RHBDL2-knockdown and control cells. First, we identified the top 20 downregulated lipid metabolism-related genes from 148 samples with concurrent DIA_DEGs, LIPID_GENEs, and IP-MS data (Fig. 2b). Second, RNA-seq analysis of these 20 lipid metabolism-related genes revealed 9 genes with no significant difference in RNA expression (*P* > 0.05 or |log2FC | < 0.5) (Supplementary Fig. 1b–e, Table S2). Finally, we evaluated the prognostic relevance of these genes in OS patients (Supplementary Fig. 1f). Notably, five candidate genes (GK5, UBE2I, PSAP, ME1, and PPT1) were prioritized. We focused on PPT1 for further investigation due to its localization in chromosome 1q, a frequently amplified genomic region widely reported in OS.

To validate the role of PPT1 in lipid metabolism in osteosarcoma (OS) cells, we first performed gain-of-function experiments by transfecting MG-63 and Saos-2 cells with OE-PPT1. Successful overexpression of PPT1 was confirmed by Western blot and RT-qPCR (Fig. 2c; Supplementary Fig. 2c–d). Consequently, intracellular levels of triglycerides, cholesterol, and free fatty acids were significantly elevated in OE-PPT1 cells compared to vector controls (Fig. 2d; Supplementary Fig. 2e). Furthermore, BODIPY493/503 staining revealed increased lipid droplet accumulation in OE-PPT1 cells, an observation confirmed by quantification (Fig. 2e; Supplementary Fig. 2f). Conversely, to explore the functional necessity of PPT1, we performed loss-of-function experiments. Efficient knockdown of PPT1 was verified in cells transfected with shPPT1-1 and shPPT1-2 (Fig. 2f; Supplementary Fig. 2b, g, h). Suppression of PPT1 led to significant reductions in triglyceride, cholesterol, and free fatty acid levels (Fig. 2g; Supplementary Fig. 2i) and a marked decrease in lipid droplet accumulation (Fig. 2h).

To clarify the part that RHBDL2 plays in modifying the lipid metabolism in OS cells via PPT1, we carried out a series of experiments. Overexpression of RHBDL2 in MG-63 and Saos-2 cells increased both RHBDL2 and PPT1 protein levels compared to the vector control (Fig. 2i; Supplementary Fig. 2k). Additionally, RHBDL2 knockdown reduced PPT1 protein expression (Fig. 2j; Supplementary Fig. 2l). The quantification of lipid components verified that PPT1 knockdown attenuated the effects of RHBDL2 overexpression on lipid metabolism (Fig. 2k; Supplementary Fig. 2m). Moreover, in cells co-transfected with OE-RHBDL2 and shPPT1, lipid droplet analysis showed reduced accumulation compared to OE-RHBDL2 with shNC (Fig. 2l; Supplementary Fig. 2n). Taken together, these findings indicate that RHBDL2 modulates lipid metabolism in osteosarcoma cells through PPT1, thus presenting a potential therapeutic avenue for osteosarcoma treatment.

### PPT1 fuels osteosarcoma malignancy by orchestrating FASN-dependent de novo lipogenesis

**Fig. 3:**
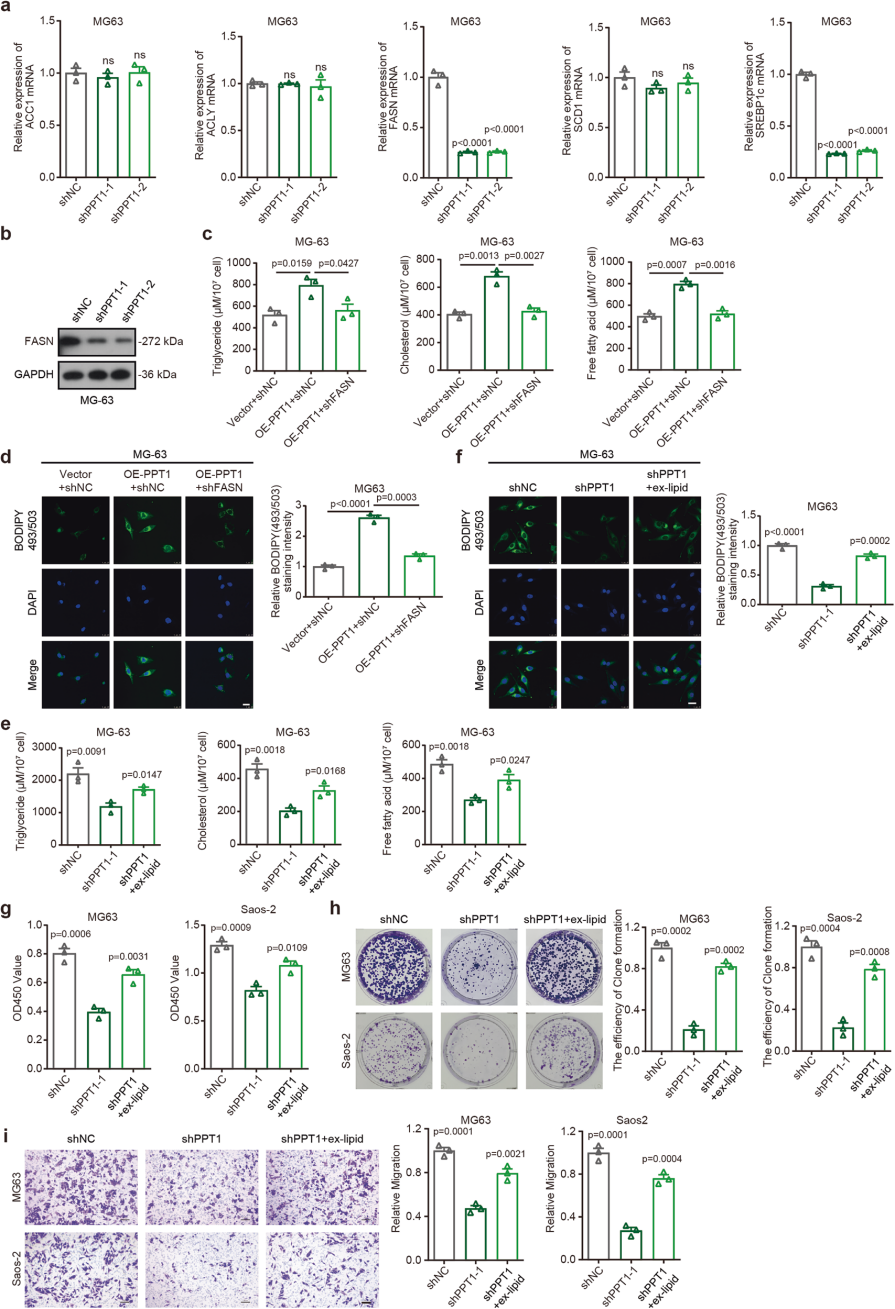
PPT1 promotes de novo lipogenesis via FASN to sustain osteosarcoma cell malignancy. **a** Relative mRNA expression of ACC1, ACLY, FASN, SCD1, and SREBP1c in MG-63 cells transfected with shNC, shPPT1-1, or shPPT1-2. **b** Western blot analysis of FASN protein levels in PPT1-knockdown MG-63 cells. **c** Quantification of triglyceride, cholesterol, and free fatty acid levels in MG-63 cells co-transfected with vector or OE-PPT1 and shFASN. Data shown as mean ± SD (*n* = 3 biologically independent experiments); unpaired *t*-test for significance. **d** Lipid droplet analysis in MG-63 cells co-transfected with vector or OE-PPT1 and shFASN. Scale bar 25 μm. Data shown as mean ± SD (*n* = 3 biologically independent experiments); unpaired *t*-test for significance. MG-63 and Saos-2 OS cells were transfected with a negative control shRNA, an shRNA targeting PPT1, or shPPT1 followed by supplementation with an exogenous lipid mixture (shPPT1+ex-lipid). **e** Quantification of triglyceride, cholesterol, and free fatty acid levels in MG-63 cells. **f** Lipid droplet imaging (BODIPY 493/503 staining, DAPI nuclei) and quantification in MG-63 cells. Scale bar 25 μm. **g** Cell viability of MG-63 and Saos-2 cells measured by CCK-8 assay. **h** Colony formation and quantification in MG-63 and Saos-2 cells. **i** Cell migration and quantification determined by Transwell assay in MG-63 and Saos-2 cells. Scale bar 100 μm. Data shown as mean ± SD (n = 3 biologically independent experiments); unpaired *t*-test for significance.

To delineate the metabolic underpinnings of PPT1-driven progression, we first profiled key enzymes of the de novo lipogenic pathway. PPT1 silencing in MG-63 and Saos-2 cells selectively precipitated a robust downregulation of FASN and SREBP1c at the transcript level, while other markers such as ACC1, ACLY, and SCD1 remained largely unaffected (Fig. 3a and Supplementary Fig. 3a). This suppression was further substantiated at the protein level, where FASN expression was markedly attenuated following PPT1 knockdown (Fig. 3b and Supplementary Fig. 3b). To establish the functional necessity of this axis, we performed epistasis experiments; notably, the exuberant accumulation of triglycerides, cholesterol, free fatty acids, and neutral lipid droplets induced by PPT1 overexpression was effectively neutralized by the concomitant silencing of FASN (Fig. 3c, d and Supplementary Fig. 3c, d). This identifies FASN as the primary metabolic effector through which PPT1 sustains the intracellular lipid reservoir.

Given that lipid scarcity often constrains rapid tumor growth, we further investigated whether the oncogenic deficits in PPT1-depleted cells were a direct consequence of metabolic starvation. Crucially, the exhaustion of lipid stores and the subsequent impairment of cellular viability, clonogenic potential, and migratory capacity triggered by PPT1 depletion were significantly salvaged—though not entirely fully restored—by the supplementation of an exogenous lipid mixture (Fig. 3e–i and Supplementary Fig. 3e, f). Collectively, these findings indicate that PPT1 serves as a metabolic rheostat, ensuring a robust FASN-mediated lipogenic flux to meet the anabolic and functional demands of osteosarcoma malignancy, a requirement that can be partially bypassed by restoring lipid availability from the extracellular environment.

### RHBDL2 stabilizes PPT1 by interacts with the deubiquitinase USP3

**Fig. 4:**
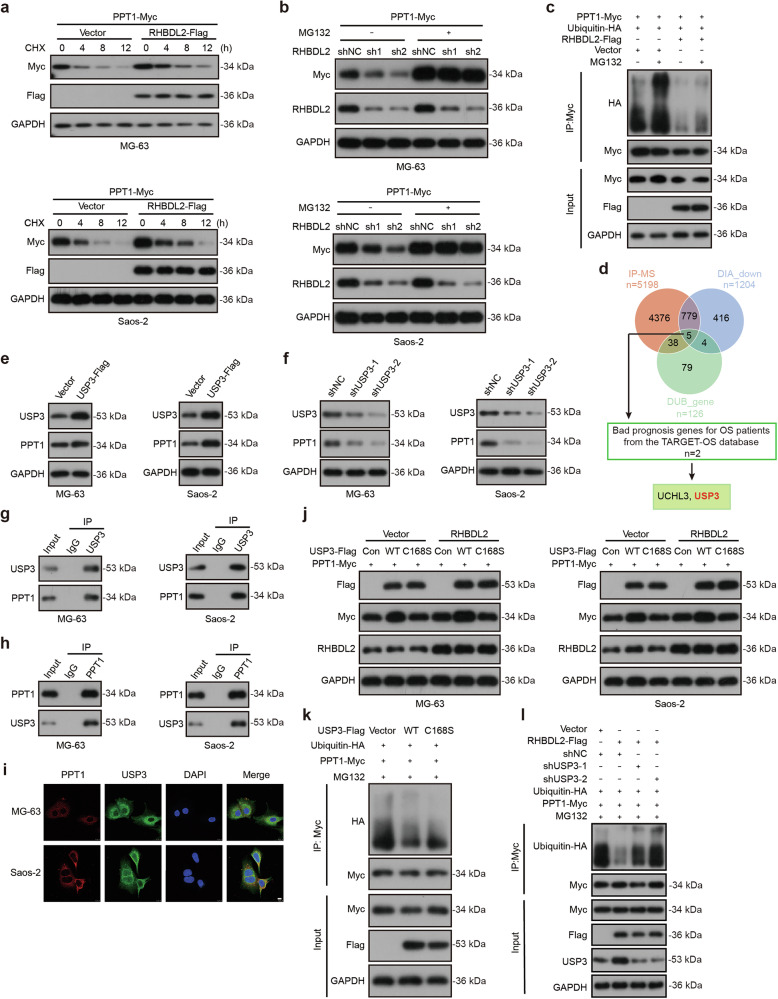
RHBDL2 prevents PPT1 degradation by mediating its deubiquitination by USP3. **a** Western blot analysis of PPT1-Myc expression in MG-63 and Saos-2 cells with RHBDL2 overexpression, after treated with cycloheximide for the indicated time. **b** Western blot analysis of PPT1-Myc expression in MG-63 and Saos-2 cells with RHBDL2 knockdown, with or without MG132 treatment. **c** PPT1-Myc and ubiquitin-HA were co-expressed with vector or RHBDL2-Flag in HEK293T cells, with or without MG132 treatment. IP was performed using Myc antibody. **d** A schematic of the screening workflow to identify the deubiquitinase USP3 as a protein candidate upregulating PPT1. Western blot analysis shows USP3 and PPT1 expression in MG-63 and Saos-2 cells with USP3 overexpression (**e**) or knockdown (**f**). **g**, **h** Co-IP showing USP3 interaction with PPT1. **i** Confocal microscopy shows PPT1 (red) and USP3 (green) colocalization in MG-63 cells, marked by DAPI (blue); merged yellow fluorescence indicates overlap. Scale bar 10 μm. **j** Western blot analysis of PPT1 expression in MG-63 and Saos-2 cells transfected with vector, RHBDL2 construct, USP3-Flag WT or USP3-Flag catalytic mutant (C168S). **k** PPT1-Myc and ubiquitin-HA were co-expressed with vector, USP3-Flag WT or USP3-Flag catalytic mutant (C168S) in HEK293T cells. After MG132 treatment, IP was performed using Myc antibody. **l** PPT1-Myc and ubiquitin-HA were co-expressed with vector or RHBDL2-Flag in USP3-knockdown cells. After MG132 treatment, IP was performed using Myc antibody.

Next, we investigated the specific molecular mechanism by which RHBDL2 regulates PPT1. We examined the mRNA expression level of PPT1 in MG-63 and Saos-2 cells under conditions of RHBDL2 overexpression or knockdown, and determined that it remained unaffected by RHBDL2 expression levels (Supplementary Fig. 4a, b). Considering that RHBDL2 affects the proteasome pathway (Fig. 2a), we treated cells with CHX and MG132 and detected the expression of PPT1 by western blot, which has clarified whether PPT1 is degraded by the proteasome pathway. The stability of PPT1-Myc was assessed in cells treated with CHX, showing that RHBDL2 overexpression affected PPT1 protein stability over time (Fig. 4a). Additionally, proteasome inhibition with MG132 totally restored PPT1 levels in RHBDL2-knockdown cells, suggesting RHBDL2 regulates PPT1 stability via ubiquitin-proteasome pathways (Fig. 4b). Ubiquitination assay revealed that RHBDL2 overexpression reduced PPT1 ubiquitination, indicating a stabilizing effect on PPT1 (Fig. 4c). Currently, there is no evidence to support the claim that RHBDL2 functions as a deubiquitinating enzyme. We hypothesize that RHBDL2 modulates the stability of PPT1 by regulating a specific ubiquitin ligase or deubiquitinating enzyme.

To identify ubiquitinating or deubiquitinating enzymes regulated by RHBDL2 that affect PPT1 stability in osteosarcoma, we analyzed multi-omics data from RHBDL2-knockdown and control cells. From this analysis, we identified five deubiquitinating enzymes using the concurrent DIA_down, DUB_gene, and IP-MS data (Fig. 4d), and forty-four ubiquitinating enzymes using the concurrent DIA_up, UB_gene, and IP-MS data (Supplementary Fig. 1g). However, due to the large number of candidate ubiquitinating enzymes, we focused our investigation on the deubiquitinating enzymes (Table S2). We then assessed the prognostic relevance of these deubiquitinating enzymes in OS patients (Supplementary Fig. 1h). Notably, two candidates, UCHL3 and USP3, were prioritized. While USP3 has been previously implicated in osteosarcoma progression [15], we focused on USP3 for further investigation due to its potential role in modulating PPT1 stability.

Next, we confirmed that PPT1 is a substrate for USP3. Western blot analysis showed that USP3 overexpression increased PPT1 levels in MG-63 and Saos-2 cells (Fig. 4e). Conversely, USP3 knockdown resulted in decreased PPT1 expression (Fig. 4f). Co-IP and IF experiments confirmed the interaction between USP3 and PPT1 (Fig. 4g–i). RHBDL2-mediated PPT1 stabilization was dependent on USP3 activity: USP3 wild-type (WT) rescued PPT1 levels, while the C168S mutant abolished this effect (Fig. 4j). In HEK293T cells, co-expression of PPT1-Myc and ubiquitin-HA with USP3-Flag WT or a catalytic mutant (C168S) revealed that USP3 WT reduced PPT1 ubiquitination, whereas the mutant did not, indicating that USP3’s deubiquitinating activity is essential for PPT1 stabilization (Fig. 4k). In USP3-depleted cells, RHBDL2 overexpression no longer suppressed PPT1 ubiquitination, underscoring USP3 as the critical mediator (Fig. 4l).

### RHBDL2 functions as a non-proteolytic scaffold to stabilize USP3 and drive lipid metabolic reprogramming

**Fig. 5:**
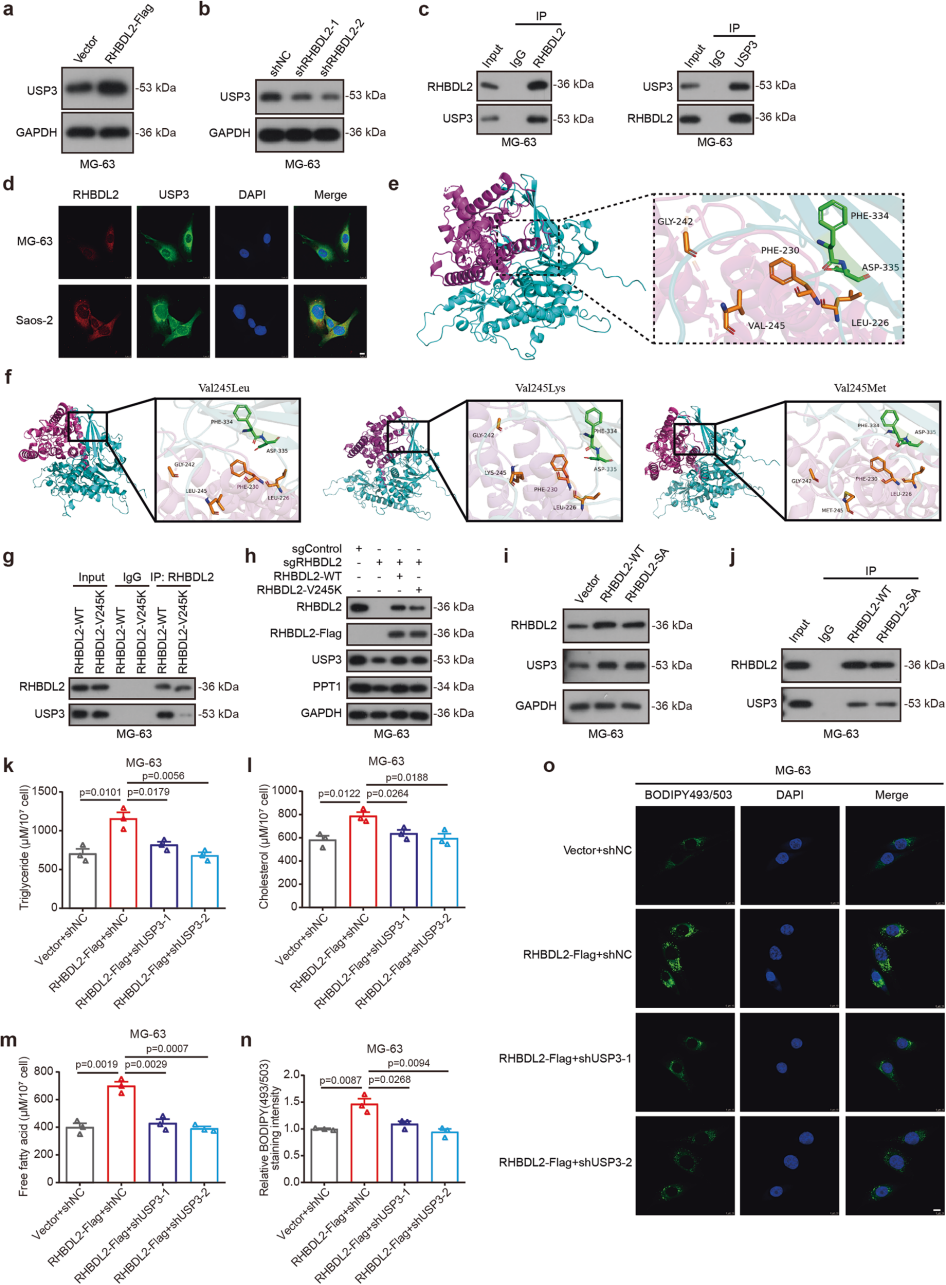
RHBDL2 acts as a scaffold to mediate its interaction with USP3 and regulate lipid metabolism. Western blot analysis shows USP3 expression in MG-63 cells with RHBDL2 overexpression (**a**) or knockdown (**b**). **c** Co-IP showing USP3 interaction with RHBDL2. **d** Confocal microscopy shows RHBDL2 (red) and USP3 (green) colocalization in MG-63 and Saos-2 cells, marked by DAPI (blue); merged yellow fluorescence indicates overlap. Scale bar 10 μm. **e** The molecular docking model of RHBDL2 (purple) and USP3 (blue) shows their interaction via a “lock-and-key” mechanism. The left panel displays the overall complex structure, while the right panel highlights critical amino acid residues (RHBDL2 in orange, USP3 in green). **f** Structural models of the USP3 (blue) and RHBDL2 (purple) binding interface, illustrating the impact of RHBDL2 Val245 mutations (Leu, Lys, Met). **g** Co-IP assay showing the interaction between RHBDL2 and USP3 in MG-63 cells. RHBDL2-V245K mutation significantly reduces binding to USP3 compared to RHBDL2-WT. **h** Western blot analysis of RHBDL2, Flag-tagged RHBDL2 variants, USP3, and PPT1 expression in MG-63 cells across the indicated groups: sgControl, sgRHBDL2, sgRHBDL2 + RHBDL2-WT, and sgRHBDL2 + RHBDL2-V245K. **i** Western blot analysis of RHBDL2 and USP3 protein expression in cells transfected with a control vector, wild-type RHBDL2 (OE-RHBDL2-WT), or the RHBDL2-SA mutant (OE-RHBDL2-SA). **j** Co-IP demonstrating the interaction of USP3 with wild-type (WT) and SA-mutant RHBDL2, contrasted with Input and IgG controls. Quantification of triglyceride (**k**), cholesterol (**l**), and free fatty acid levels (**m**) in MG-63 cells co-transfected with vector or RHBDL2-Flag and shNC or shUSP3-1/-2. Data shown as mean ± SD (*n* = 3 biologically independent experiments); unpaired *t*-test for significance. **n** Lipid droplet quantification in MG-63 cells co-transfected with Vector or RHBDL2-Flag and shNC or shUSP3-1/-2. Data shown as mean ± SD (*n* = 3 biologically independent experiments); unpaired *t*-test for significance. **o** Lipid droplet (BODIPY493/503 staining, DAPI nuclei) imaging in MG-63 cells co-transfected with vector or RHBDL2-Flag and shNC or shUSP3-1/-2. Scale bar 10 μm.

To further define the biochemical nature of the RHBDL2-USP3 interaction, we investigated whether RHBDL2’s protease activity was required for this regulatory effect. Western blot analysis confirmed that USP3 expression was positively regulated by RHBDL2 (Fig. 5a, b and Supplementary Fig. 4c). Co-IP and IF experiments confirmed the interaction between USP3 and RHBDL2 (Fig. 5c, d and Supplementary Fig. 4d). To elucidate the structural basis of this interaction, the rigid protein-protein docking model revealed that residues such as PHE-334 and ASP-335 of RHBDL2 were embedded within the hydrophobic pocket of USP3 through hydrophobic interactions, with stabilization further reinforced by π-π stacking. Importantly, VAL-245, together with LEU-226, contributed to the formation of a compact hydrophobic core, while the inherent flexibility of GLY-242 suggested conformational adaptation during binding (Fig. 5e). To validate the critical role of VAL-245, we introduced point mutations at this site. Structural modeling predicted that mutations to Leu, Lys, or Met would all disrupt the interaction, albeit through distinct mechanisms: Val245Leu introduced steric hindrance with USP3’s PHE-230; Val245Lys, being a charged residue, fundamentally disrupted the hydrophobic core; and Val245Met, while still hydrophobic, altered the core’s geometry (Fig. 5f). Critically, Co-IP experiments validated a interaction between USP3 and RHBDL2 and confirmed that the RHBDL2-V245K mutation, as a representative, significantly impaired this binding (Fig. 5g and Supplementary Fig. 4e). Furthermore, reconstitution of RHBDL2-knockout cells with the V245K mutant failed to fully restore USP3 and PPT1 protein levels, unlike the wild-type RHBDL2 (Fig. 5h and Supplementary Fig. 4f), consolidating the mechanistic link between RHBDL2’s Val245 residue and USP3 stability. To determine if this regulation required the catalytic activity of RHBDL2, we generated a protease-dead mutant, RHBDL2-SA. Overexpression of either the WT or SA mutant led to a comparable increase in USP3 protein levels (Fig. 5i and Supplementary Fig. 4g). Furthermore, co-immunoprecipitation assays revealed that the RHBDL2-SA mutant interacted with USP3 as robustly as its wild-type (WT) counterpart (Fig. 5j and Supplementary Fig. 4h). These results demonstrate that RHBDL2 stabilizes USP3 through a scaffolding mechanism, independent of its protease activity. This structural requirement translated directly to metabolic control; while RHBDL2 overexpression triggered a profound surge in intracellular lipids and lipid droplet expansion, these effects were largely abrogated upon USP3 silencing (Fig. 5k–o and Supplementary Fig. 4i–m).

Having established that the VAL-245 residue is critical for the RHBDL2-USP3 interaction and subsequent PPT1 stabilization, we hypothesized that the V245K mutant would be deficient in promoting the associated oncogenic phenotypes. Consistent with the reduced PPT1 expression (Fig. 3), the RHBDL2-V245K mutant was significantly impaired in its ability to promote intracellular lipid accumulation. Cells expressing the V245K mutant exhibited lower levels of neutral lipids (BODIPY staining), triglycerides, cholesterol, and free fatty acids compared to those reconstituted with RHBDL2-WT (Supplementary Fig. 5a–d). Functionally, this mutation also blunted the pro-tumorigenic phenotypes. The RHBDL2-V245K mutant was compromised in rescuing the proliferation (CCK-8 and colony formation) and migration (Transwell and wound healing) deficits observed in RHBDL2-knockout cells, performing significantly worse than the WT rescue (Supplementary Fig. 5e–i). These results collectively demonstrate that RHBDL2 interacts with USP3 via a critical hydrophobic core anchored by VAL-245 to regulate PPT1 stability in osteosarcoma cells. The functional impairment caused by the V245K mutant across all downstream phenotypes underscores the essential nature of this interaction, highlighting a novel pathway involving RHBDL2, USP3, and PPT1 in osteosarcoma pathogenesis.

### RHBDL2 drives OS progression via USP3-mediated regulation of proliferation, migration, and tumor growth

**Fig. 6:**
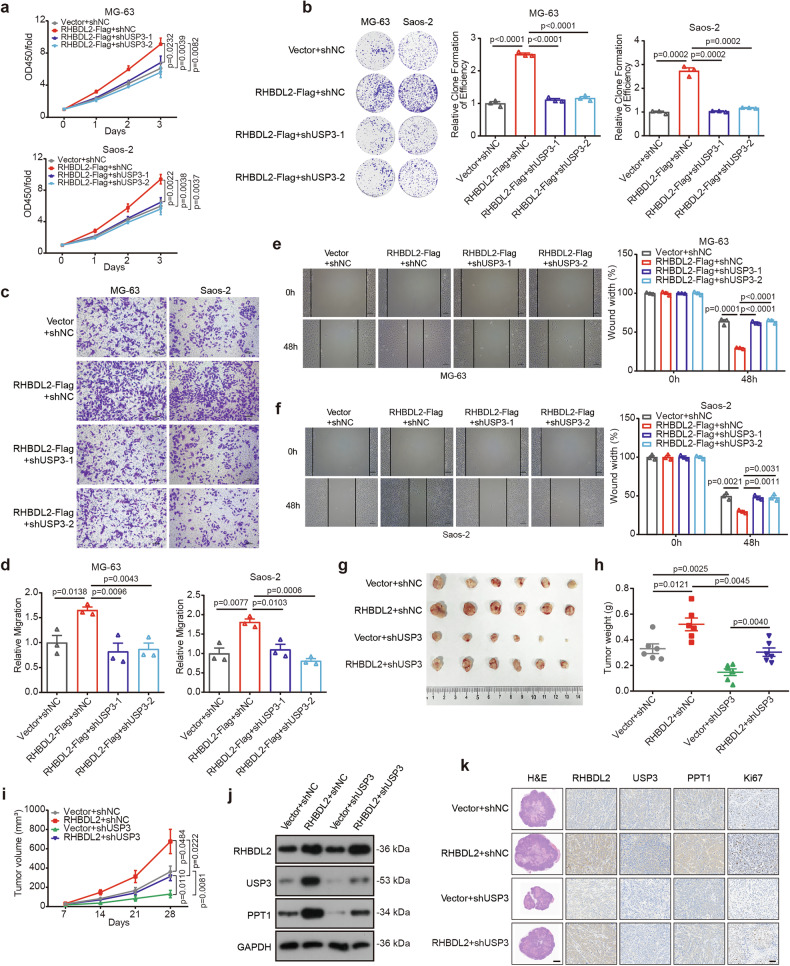
RHBDL2 promotes osteosarcoma progression via USP3-mediated PPT1 stabilization. **a** CCK-8 assay showing the effect of RHBDL2 overexpression and USP3 knockdown on the proliferation of OS cells over 3 days. Data shown as mean ± SD (*n* = 3 biologically independent experiments); unpaired *t*-test for significance. **b** Colony formation assay demonstrating the impact of RHBDL2 and USP3 on the clonogenic ability of OS cells. Representative images of colonies stained with crystal violet are shown. Data shown as mean ± SD (*n* = 3 biologically independent experiments); unpaired *t*-test for significance. **c**, **d** Transwell migration assay indicating the role of RHBDL2 and USP3 in the migratory potential of OS cells. Representative images of stained cells are shown. Scale bar 100 μm. Data shown as mean ± SD (*n* = 3 biologically independent experiments); unpaired *t*-test for significance. Wound healing assay illustrating the effect of RHBDL2 and USP3 on cell migration in MG-63 (**e**) and Saos-2 (**f**) cells. Representative images of wound closure at 0 h and 48 h post-scratch are shown. Scale bar 100 μm. Data shown as mean ± SD (*n* = 3 biologically independent experiments); unpaired *t*-test for significance. **g** Xenograft tumor model showing the effect of RHBDL2 and USP3 on tumor growth in vivo. Representative images of tumors are shown. Tumor weight (**h**) and volume (**i**) measurements from the xenograft model. Data shown as mean ± SD (*n* = 6 in each group); unpaired *t*-test for significance. **j** Western blot analysis of the indicated proteins, normalized to GAPDH, was performed on OS tissues from mice in Vector + shNC, RHBDL2 + shNC, Vector + shUSP3, and RHBDL2 + shUSP3 groups. **k** H&E staining and IHC analysis of tumor tissues for RHBDL2, USP3, PPT1, and Ki67 expression. Representative images are shown. The scale bars are 1250 μm and 50 μm, respectively.

To investigate the role of RHBDL2 and USP3 in OS progression, we first assessed their impact on cell proliferation. CCK-8 assays demonstrated that RHBDL2 overexpression significantly enhanced OS cell proliferation over 3 days compared to vector controls, while USP3 knockdown in RHBDL2-overexpressing cells attenuated this effect (Fig. 6a). The colony formation assay further confirmed these findings, showing that RHBDL2 overexpression increased the clonogenic ability of OS cells, while USP3 knockdown reduced the number and size of colonies formed (Fig. 6b). Next, we evaluated the role of RHBDL2 and USP3 in OS cell migration. The Transwell assay revealed that RHBDL2 overexpression significantly increased the migratory potential of MG-63 and Saos-2 cells compared to vector controls (Fig. 6c, d). However, this enhanced migration was markedly reduced by USP3 knockdown. The wound healing assay corroborated these results, showing faster wound closure in RHBDL2-overexpressing cells, which was inhibited by USP3 knockdown (Fig. 6e, f).

Additionally, the results of flow cytometry analysis indicated that RHBDL2 knockdown significantly increased the apoptotic rate in both cell lines compared to the shNC group (Supplementary Fig. 6a). Further results demonstrated that RHBDL2 overexpression significantly reduced apoptosis in both cell lines. However, this anti-apoptotic effect was attenuated by USP3 knockdown, indicating that USP3 is essential for RHBDL2’s regulation of apoptosis (Supplementary Fig. 6b). It is reported that USP3 promotes osteosarcoma progression via activating the PI3K-AKT signaling pathway [15]. Western blot analyses were performed to examine the expression levels of proteins involved in the PI3K-AKT signaling pathway and EMT in MG-63 and Saos-2 cells (Supplementary Fig. 6c, d). For the PI3K-AKT pathway, the results showed that RHBDL2 knockdown reduced the phosphorylation levels of PI3K and AKT, indicating inhibited pathway activation. Conversely, RHBDL2 overexpression increased PI3K and AKT phosphorylation, which was reversed by USP3 knockdown.

Regarding EMT markers, RHBDL2 knockdown led to increased expression of the epithelial marker E-cadherin and decreased expression of the mesenchymal markers N-cadherin, Snail, Slug, and ZEB1 in both cell lines. This suggests that RHBDL2 promotes EMT. Conversely, RHBDL2 overexpression enhanced the expression of mesenchymal markers and reduced E-cadherin levels. USP3 knockdown in RHBDL2-overexpressing cells restored E-cadherin expression and reduced mesenchymal marker levels, indicating that USP3 is crucial for RHBDL2’s regulation of EMT. These results demonstrate that RHBDL2 plays a significant role in regulating apoptosis, the PI3K-AKT signaling pathway, and EMT in osteosarcoma cells.

To validate these findings in vivo, we established a xenograft tumor model. Tumors from RHBDL2-overexpressing cells exhibited significantly greater volume and weight compared to vector control tumors (Fig. 6g–i). USP3 knockdown in RHBDL2-overexpressing cells led to a reduction in tumor growth, indicating that USP3 is essential for RHBDL2-driven tumor progression. Western blot analysis, H&E staining and IHC analysis of tumor tissues revealed elevated expression of RHBDL2, USP3, PPT1, and the proliferation marker Ki67 in RHBDL2-overexpressing tumors (Fig. 6j, k). USP3 knockdown reduced the expression levels of these proteins, consistent with the functional roles observed in vitro. These comprehensive results demonstrate that RHBDL2 promotes osteosarcoma progression through USP3-mediated stabilization of PPT1. This study identifies the RHBDL2-USP3-PPT1 axis as a potential therapeutic target for osteosarcoma treatment, offering new insights into the mechanisms underlying osteosarcoma progression.

### Pharmacological targeting of the RHBDL2-USP3 interaction with EGCG suppresses osteosarcoma progression

**Fig. 7:**
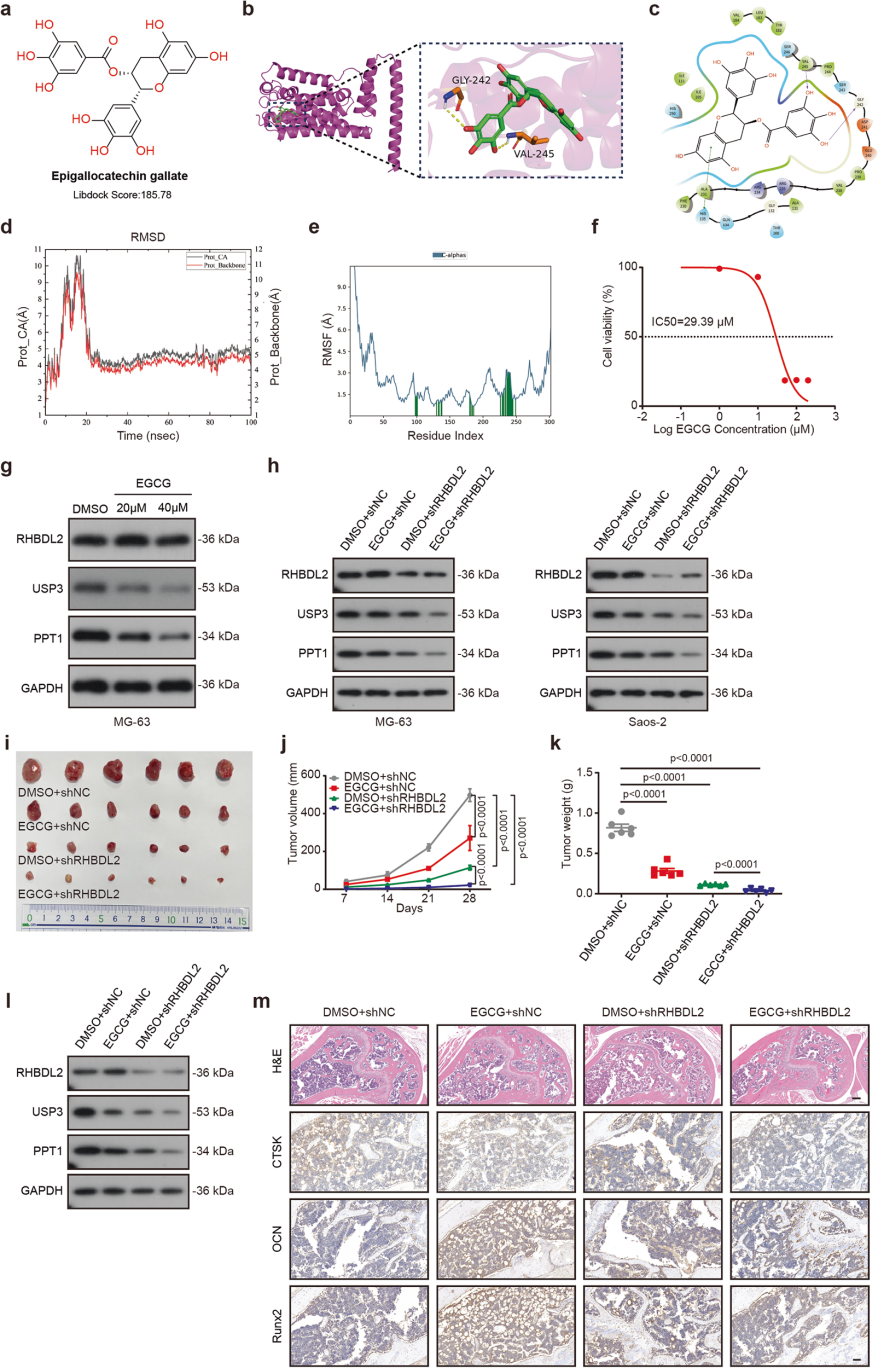
EGCG targeting the interaction of RHBDL2 and USP3. **a** The chemical structures and docking scores of EGCG are presented. **b** Global views of the complexes formed by EGCG are depicted. **c** Detailed views of the complexes formed by EGCG are shown. The dynamics of the complexes of EGCG with RHBDL2 are illustrated by RMSD (**d**) and RMSF (**e**) plots. **f** CCK-8 assay was employed to evaluate the inhibitory effects of EGCG on osteosarcoma cells. **g** Western blot analysis of RHBDL2, USP3, and PPT1 expression in MG-63 cells treated with EGCG for the indicated concentration. **h** Western blot analysis of RHBDL2, USP3, and PPT1 expression in MG-63 and Saos-2 cells transfected with control shRNA or RHBDL2 shRNA and subsequently treated with either DMSO or EGCG. **i**–**k** An in vivo xenograft model was established using MG-63 cells stably expressing shNC or shRHBDL2. Mice were treated with vehicle (DMSO) or EGCG (50 mg/kg/day, by oral gavage). Representative images of excised tumors at the study endpoint (**i**). Tumor growth curves (**j**) and final tumor weights (**k**). Data shown as mean ± SD (*n* = 6 in each group); unpaired *t*-test for significance. **l** Western blot analysis of RHBDL2, USP3, and PPT1 protein levels in representative tumor lysates from each group. **m** Histological analysis of bone tissue from the tumor-bearing limbs, showing representative images of Hematoxylin and Eosin (H&E) staining for tissue morphology, and IHC for the bone markers Cathepsin K (CTSK), Osteocalcin (OCN), and Runx2.

To identify small-molecule inhibitors targeting the RHBDL2/USP3 interaction interface, we performed molecular docking simulations based on the predicted binding pocket surrounding the critical residue VAL-245 (Fig. 5e). High - throughput docking screening against a small - molecule compound library yielded five candidates: Imatinib, Luteolin 3′-O-β-D-glucuronide, Epigallocatechin gallate (EGCG), Calceolarioside B, and Baicalin methyl ester (Table S3). RMSD curves showed all five compound-protein complexes were stable within 100 ns (Fig. 7d; Supplementary Fig. 7d). Docking results revealed Imatinib had a score of 185.78 and interaction energy of 74.054 kcal/mol, forming hydrogen bonds with RHBDL2’s VAL-184, SER-187, ASP-227, ARG-234, SER-246, and HIS-250. Luteolin 3′-O-β-D-glucuronide had a score of 184.293 and interaction energy of 66.67 kcal/mol, bonding with GLN-134, ASN-139, and ARG-234. EGCG (score 182.261, energy 50.75 kcal/mol) interacted with GLY-242 and VAL-245. Calceolarioside B (score 180.894, energy 56.95 kcal/mol) formed hydrogen bonds with GLN-134, HIS-135, VAL-184, SER-187, SER-246, and HIS-250. Baicalin methyl ester (score 174.913, energy 69.42 kcal/mol) bonded with GLN-134, ASN-139, ASP-227, and ARG-234 (Fig. 7a–e; Supplementary Fig. 7a–e). Functional validation in osteosarcoma cells demonstrated dose-dependent anti-proliferative effects, with EGCG exhibiting the highest potency (IC = 29.39 μM), followed by Imatinib (37.39 μM), Baicalin methyl ester (38.88 μM), Calceolarioside B (43.87 μM), and Luteolin 3′-O-β-D-glucuronide (209.0 μM) (Fig. 7f; Supplementary Fig. 7f). Moreover, EGCG decreased the protein level of USP3, and PPT1 in a dose-dependent manner (Fig. 7g). These results nominate EGCG as a lead candidate for disrupting the RHBDL2/USP3 axis.

To validate the on-target activity and therapeutic potential of EGCG, we conducted further in vitro and in vivo experiments. First, we confirmed that EGCG’s effect was dependent on its intended target. The ability of EGCG to downregulate USP3 and PPT1 was significantly attenuated in RHBDL2-knockdown cells, confirming that its mechanism of action is RHBDL2-dependent (Fig. 7h). Encouraged by these findings, we assessed the efficacy of EGCG in an xenograft mouse model using MG-63 cells. EGCG treatment significantly suppressed tumor growth, as evidenced by reduced tumor volume and weight compared to the vehicle-treated group (Fig. 7i–k). Importantly, this anti-tumor effect was substantially diminished in mice bearing shRHBDL2 tumors, providing strong in vivo evidence for on-target engagement. Western blot analysis of tumor lysates confirmed that EGCG inhibited the RHBDL2-USP3-PPT1 axis in vivo (Fig. 7l). Furthermore, histological analysis of adjacent bone tissue revealed that EGCG treatment mitigated tumor-induced bone destruction, as indicated by preserved trabecular architecture (H&E staining) and a shift towards a pro-osteogenic state with reduced osteoclast activity (increased Runx2/OCN, decreased CTSK) (Fig. 7m).

EGCG binds to the RHBDL2 catalytic pocket through a multimodal mechanism:(1) A hydrogen-bonding network involving hydroxyl groups with GLN-134 (occupancy: 68%), SER-246 (48%), HIS-135 (36%), and water-mediated interactions near ASP-227; (2) Hydrophobic packing with VAL-184 and ILE residues; (3) Potential π-π stacking with TYR-182 (48%). GLN-134 serves as a critical anchor, with water-bridged hydrogen bonds enhancing binding stability (Fig. 7b, c). Molecular dynamics simulations revealed stable complex formation (RMSD: 1.5-2.0 Å) and localized rigidity at the binding site (RMSF < 1.5 Å for VAL-245 and GLY-242), contrasting with higher flexibility in distal regions (RMSF ~ 3.0 Å for residues 50-150) (Fig. 7d, e). This rigidity indicates EGCG may competitively inhibit RHBDL2’s interaction with its substrate USP3 by restricting conformational changes at the substrate-binding site. The synergistic interplay of hydrogen bonding, hydrophobic forces, and structural stabilization positions EGCG as a promising inhibitor of the RHBDL2/USP3 interface, with therapeutic potential to disrupt osteosarcoma progression.

## Discussion

**Fig. 8:**
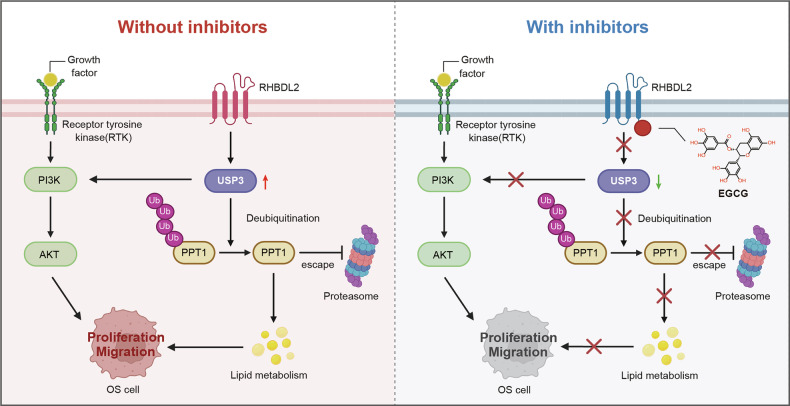
The mechanistic diagram of RHBDL2-USP3-PPT1 axis that drives lipid metabolic reprogramming and OS progression. RHBDL2 stabilizes PPT1 by upregulating USP3, which removes PPT1’s ubiquitin chains, enhancing lipid metabolism. RHBDL2 also interacts with USP3 to activate the PI3K/AKT pathway, promoting osteosarcoma cell proliferation and migration. EGCG inhibits this by competitively binding to RHBDL2, reducing USP3 expression, impairing PPT1 deubiquitination and stability, and inhibiting lipid metabolism. Lowered USP3 deactivates the PI3K/AKT pathway, curbing tumor growth and spread. The schematic was Created in BioRender.

Our study demonstrates that RHBDL2 plays a pivotal role in OS progression by driving lipid metabolic reprogramming through the USP3-mediated stabilization of PPT1. We observed that RHBDL2 is overexpressed in OS tumor tissues compared to normal tissues, correlating with poor patient prognosis. Functional assays revealed that RHBDL2 suppression significantly inhibits OS cell proliferation, migration, and lipid metabolism, while enhancing apoptosis. Mechanistically, RHBDL2 interacts with the deubiquitinating enzyme USP3, preventing PPT1 ubiquitination and degradation, thereby enhancing lipid synthesis and storage (Fig. 8).

This study unveils a novel RHBDL2-USP3-PPT1 axis driving lipid metabolic reprogramming in OS, a mechanism distinct from RHBDL2’s previously reported roles in other cancers. While prior work established RHBDL2 as a regulator of PI3K/AKT pathway in gastric cancer [8] and a suppressor of cuproptosis in renal cell carcinoma [7], our findings are the first to link RHBDL2 to lipid metabolism in OS through USP3-mediated deubiquitination of PPT1. Other studies show RHBDL2 works with OTUD7B to stabilize N1ICD via the ubiquitin - proteasome pathway, activating the Notch signaling pathway and promoting pancreatic cancer cell proliferation and migration [6]. Our study identifies RHBDL2 as a non-proteolytic scaffold that stabilizes USP3 through a hydrophobic interface anchored by the VAL-245 residue, thereby shielding PPT1 from degradation to drive OS malignancy. This interaction is independent of RHBDL2’s catalytic activity but essential for PPT1-mediated lipogenesis and tumor progression. Mechanistically, we propose that RHBDL2 binding may induce conformational changes in USP3 to mask degradation-sensitive sites, potentially complemented by a reciprocal feedback loop where USP3-mediated deubiquitination of RHBDL2 further amplifies the stability of this axis. Collectively, the RHBDL2-USP3-PPT1 signaling cascade represents a critical structural and metabolic vulnerability in osteosarcoma.

Unlike studies focusing on RHBDL2’s proteolytic activity [16], we demonstrate its non-catalytic role in stabilizing metabolic enzymes via ubiquitin-proteasome crosstalk. In addition, we observed that RHBDL2 overexpression enhanced the phosphorylation of PI3K and AKT, thereby activating the PI3K-AKT pathway and modulating key EMT transcription factors (Snail, ZEB1, Slug). Consequently, it suppressed the expression of the epithelial marker E-cadherin and promoted the expression of the mesenchymal marker N-cadherin, effects that were reversed upon USP3 knockdown. These findings are consistent with those reported by Anan Li et al. [15], who demonstrated that USP3 facilitates osteosarcoma progression via deubiquitination of EPHA2 and activation of PI3K-AKT signaling. However, RHBDL2 upregulated the expression of EMT-related transcription factors (ZEB1, SNAIL1, and TWIST1) through the Notch signaling pathway in RHBDL2-overexpressing pancreatic cancer cells [6]. The interplay between the PI3K-AKT pathway and EMT has been extensively investigated across various cancers [17–19], suggesting that the regulatory mechanisms of RHBDL2 may vary depending on the cancer type.

Furthermore, our multi-omics approach (RNA-seq, DIA proteomics, and IP-MS) identified PPT1 as a key downstream effector, addressing a critical gap in understanding how lipid metabolic enzymes are post-translationally regulated in OS. This contrasts with existing literature emphasizing transcriptional or epigenetic control of lipid metabolism (e.g., ACLY upregulation via transcription factor SIX1 [20]), highlighting the novelty of our ubiquitination-centric mechanism. PPT1 was identified as a key factor influencing the synthesis of membrane lipids and the functionality of organelles, such as lysosomes, by removing protein palmitoylation modifications [21]. The loss of PPT1 function results in the accumulation of palmitoylated proteins within lysosomes, thereby disrupting lysosomal activity and impairing lipid degradation and recycling processes. For instance, PPT1 dysfunction leads to infantile neuronal ceroid lipofuscinosis, a pathological condition marked by the buildup of lipofuscin (lipid- and protein-rich deposits) in lysosomes [22–24]. Additionally, defects in PPT1 have been shown to disturb GABA-receptor palmitoylation homeostasis, consequently affecting neuronal signaling and lipid metabolism [23]. While the direct involvement of PPT1 in lipid homeostasis has remained largely elusive, our study identifies it as a pivotal orchestrator of de novo lipogenesis in OS cells. We demonstrate that PPT1 deficiency selectively precipitates a robust downregulation of the FASN/SREBP1c axis, leading to the profound depletion of intracellular triglycerides, cholesterol, and free fatty acids. Crucially, the metabolic surge and exuberant lipid droplet accumulation driven by PPT1 overexpression are functionally contingent upon FASN activity, as evidenced by the effective neutralization of these effects upon FASN silencing. Beyond mere metabolic maintenance, this PPT1-FASN circuitry is indispensable for sustaining the malignant phenotype; the marked impairments in cellular viability, clonogenic potential, and migratory capacity triggered by PPT1 loss are significantly salvaged by the restoration of lipid availability through exogenous supplementation. Collectively, these findings establish PPT1 as a metabolic rheostat that fuels OS malignancy by ensuring a robust FASN-dependent lipogenic flux to meet the anabolic demands of tumor progression.

The development of selective inhibitors that disrupt oncogenic lipid metabolism while preserving physiological bone remodeling remains a major challenge in osteosarcoma therapy. Current mTORC1 inhibitors (e.g., rapamycin) suppress lipogenesis but impair growth plate chondrocyte differentiation, leading to skeletal growth arrest [25–28]. Through high-throughput screening, we identified EGCG—a flavan-3-ol polyphenol with eight free hydroxyl groups and the most abundant catechin in green tea (~50% of total polyphenols) [29]—as a candidate inhibitor. EGCG exhibits potent antioxidant activity via free radical scavenging (e.g., DPPH) and suppression of oxidative stress enzymes (e.g., NADPH oxidase) [29–31], alongside anti-inflammatory effects through modulation of TNF-α, IL-6, and NF-κB signaling [32–34]. Preclinical studies highlight its broad anticancer potential, including inhibition of proliferation, angiogenesis, and apoptosis induction via MAPK and PI3K/AKT pathways [35–39]. Mechanistically, EGCG binds to RHBDL2, competitively blocking its interaction with USP3, reducing USP3 stability, and consequently affecting PPT1 stability and lipid metabolism, thus curbing tumor development. We confirmed that EGCG’s capacity to suppress the USP3-PPT1 axis and inhibit tumor growth is strictly RHBDL2-dependent, as evidenced by its diminished efficacy in RHBDL2-knockdown models. Furthermore, EGCG mitigated tumor-induced bone destruction, preserving trabecular architecture and promoting a pro-osteogenic microenvironment characterized by elevated Runx2/OCN levels and suppressed osteoclast activity, as evidenced by decreased CTSK expression. These findings are particularly significant in the context of the bone microenvironment, as previous research has shown that RHBDL2 is a protease that cleaves Thrombomodulin’s (TM) transmembrane domain, causing the release of its extracellular domain. In osteoid MG-63 cells, mechanical injury upregulates RHBDL2, facilitating TM cleavage and sTM secretion; subsequently, sTM’s TMD2/3 promotes osteocyte functions and bone healing by activating the FGFR/ERK signaling pathway. Given that RHBDL2 activity is a key upstream regulator of these processes [40], the dual role of EGCG in inhibiting pathological tumor progression while mitigating bone destruction suggests a complex therapeutic potential in osteosarcoma. The reported IC50 of EGCG in our study is 29.39 μM, which is considerably higher than the peak plasma concentrations (Cmax ~0.16–0.27 μM) observed in humans following oral administration of green tea or pure EGCG, as documented in clinical studies [41]. This notable disparity underscores the challenge of achieving therapeutic EGCG levels in vivo through conventional oral dosing, largely due to its poor bioavailability, extensive metabolism, and rapid systemic clearance. To overcome these limitations, the use of advanced delivery systems such as nanoparticles has been proposed. For instance, nano-encapsulation of EGCG in PLA-PEG nanoparticles has been shown to enhance its cellular uptake and antitumor efficacy, allowing a 10-fold reduction in the effective dose required to induce apoptosis in cancer cells [42]. Therefore, employing nanocarriers represents a promising strategy to elevate EGCG bioavailability and achieve physiologically relevant concentrations for effective osteosarcoma therapy. These findings position EGCG as a promising therapeutic agent for osteosarcoma, though further preclinical validation and clinical studies are required to confirm efficacy and safety in humans.

## Methods

### Tissue specimens

This study was approved by the Medical Ethics Committee of the Second XIANGYA Hospital of Central South University. Informed consents were obtained from all patients for this study. We collected 50 tissue specimens of frozen in liquid nitrogen OS and corresponding paratumoral tissue specimens from the Second XIANGYA Hospital of Central South University. The IHC microarray tissues (LN020Bn01 and L072Bn01) were purchased from Bioaitech Inc. (Xi’an, China).

### Cell culture, Lentivirus infection and Plasmids

HEK293T and osteosarcoma (MG-63, Saos-2) cell lines from ATCC were cultured in DMEM (Corning, 10-013-CVR) with 10% FBS (Ausbian, VS500T) at 37 °C, 5% CO_2_. Lentiviral amplification was conducted in HEK293T cells. Viral supernatants collected at 48 and 72 h post-transfection were filtered through a 0.45-μm membrane and used to transduce target cells with 8 μg/ml polybrene. Transduced cells were selected via antibiotic resistance.

The catalytically inactive RHBDL2-SA mutant was generated by substituting the active-site serine residue with alanine [43, 44]. The RHBDL2-Flag constructs (wild-type and V245K mutant) were generated by site-directed mutagenesis, with nucleotides 733-735 (GTG) in the wild-type sequence mutated to AAG. The constructs RHBDL2-Flag (WT and V245K), PPT1-Myc, Ubiquitin-HA, USP3-Flag (WT and C168S), USP3 shRNA (shUSP3-1 and shUSP3-2), PPT1 shRNA (shPPT1-1 and shPPT1-2), RHBDL2 shRNA (shRHBDL2-1 and shRHBDL2-2), and sgRHBDL2 were generated using standard molecular biology techniques and confirmed by sequencing. Target sequences were as follows:

Control shRNA: 5′-TTCTCCGAACGTGTCACGT-3′;

shRHBDL2-1: 5′-GGAGTTGTCATAAGGGTGTTG-3′;

shRHBDL2-2: 5′-GGCGAAGAAGGGAAGGAAAG-3′;

shRHBDL2-3: 5′-CCAACACCCTTATGACAACTC-3′;

shUSP3-1: 5′-CCAACCATAAGAAATCAGAAA-3′;

shUSP3-2: 5′-GCCTCATATGTGGGACAGAAT-3′;

shPPT1-1: 5′-GCACTTGCTAAGGATCCTAAA-3′;

shPPT1-2: 5′-GCCCACATCATACCATTCCTT-3′;

sgRHBDL2: 5′-CCAAGAGTAAAAAGGTCCAC-3′;

shFASN: 5′-GGTGTGTGCTGCTCTCCAA-3′.

### Chemicals

Cycloheximide (66-81-9) was obtained from TargetMol, and the final concentration was 50 µg/mL. MG132 (133407-82-6) was obtained from TargetMol, and the final concentration was 25 μM. Imatinib (HY-15463), Luteolin 3′-O-β-D-glucuronide (HY-N4099), Epigallocatechin gallate (HY-13653), Calceolarioside B (HY-N0539), and Baicalin methyl ester (HY-N4297) were obtained from MedChemExpress. Exogenous lipid mixture: 200 μM oleate + 100 μM palmitate + 20 μM cholesterol, BSA-conjugated.

### Quantitative real-time PCR (RT-qPCR)

Total RNA was extracted from cells using TRIzol (Thermo, 15596026CN) and reverse-transcribed with ReverTra Ace qPCR RT Kit (TOYOBO, FSQ-101). qPCR was performed in triplicate using SYBR High-Sensitivity qPCR Supermix (Novoprotein, E099-01B). Relative mRNA levels were quantified using the 2^−ΔCt^ method. Primer sequences are listed below (DLM, Wuhan, China):

RHBDL2-F: 5′-TGGAGTTCAGCACATCTTGG-3′;

RHBDL2-R:5′-AATAGCCTCCCATCAGAGCA-3′;

PPT1-F:5′-ACTGGCATGACCCCATAAAG-3′;

PPT1-R: 5′-CTCCGAATCTACAGGGTCCA-3′;

USP3-F: 5′-ATACCACACCAGGAGCCAAG-3′;

USP3-R: 5′-CCAAAAGGTAGCGCATGAAT-3′;

β-Actin-F: 5′-TGGACTTCGAGCAAGAGATG-3′;

β-Actin-R: 5′-GAAGGAAGGCTGGAAGAGTG-3′;

ACC1-F: 5′-AGCCTACGCACGAAAGTGAC-3′;

ACC1-R: 5′-TGTTGGCAATCGTTTCCATATC-3′;

FASN-F: 5′-AGTACACACCCAAGGCCAAG-3′;

FASN-R: 5′-GTGGATGATGCTGATGATGG-3′;

SCD1-F: 5′-CCCAGCTGTCAAAGAGAAGG-3′;

SCD1-R: 5′-CAAGAAAGTGGCAACGAACA-3′;

ACLY-F: 5′-ATCTCCGGCCTCTTCAATTT-3′;

ACLY-R: 5′-ACTCGATGTCACCCCACTTC-3′;

SREBP1c-F: 5′-GCAGCCACCATCTAGCCTG-3′;

SREBP1c-R: 5′-CAGCAGTGAGTCTGCCTTGAT-3′.

### Coimmunoprecipitation (Co-IP) assay and Western Blot Analysis

Cells were lysed in RIPA buffer (Beyotime, P0013B) containing protease inhibitors (Sigma, P8340-1ml) and centrifuged at 12,000 × *g* for 15 min at 4 °C. The supernatant was pre-cleared with protein A/G beads for 1 h at 4 °C. The pre-cleared lysate was incubated with the primary antibody overnight at 4 °C, with a negative control lacking the primary antibody. Protein A/G beads were added to capture the antibody-protein complex and incubated for 4 h at 4 °C. The beads were washed with lysis buffer, and bound proteins were eluted by boiling in SDS-PAGE loading buffer. Proteins were resolved by SDS-PAGE on 6% or 10% gels and transferred to PVDF membranes (Millipore, IPVH00010). Membranes were incubated with primary antibodies against RHBDL2 (1:1000, Proteintech, 12467-1-AP), PPT1 (1:1000, Proteintech, 29653-1-AP), USP3 (1:1000, Proteintech, 12490-1-AP), PIK3CA (1:1000, Proteintech, 67071-1-Ig), PIK3R1 (1:1000, Proteintech, 60225-1-Ig), AKT (1:2000, Proteintech, 10176-2-AP), p-AKT(S473) (1:1000, Proteintech, 28731-1-AP), E-cadherin (1:20000, Proteintech, 20874-1-AP), N-cadherin (1:2000, Proteintech, 22018-1-AP), ZEB1 (1:1000, Proteintech, 21544-1-AP), MYC-tag (1:5000, Proteintech, 16286-1-AP), DYKDDDDK-tag (1:30000, Proteintech, 20543-1-AP), HA-tag (1:5000, Proteintech, 51064-2-AP), Slug (1:1000, CST, 9585), Snail (1:1000, CST, 3879), FASN (1:1000, Proteintech, 10624-2-AP), and GAPDH (1:30000, Proteintech, 10494-1-AP), followed by HRP-conjugated secondary antibodies.

### Ubiquitination assay

Cells were treated with MG132 (25 μM, Sigma, 474790) for 6 h, harvested in ice-cold PBS, and lysed in RIPA buffer with protease inhibitors on ice for 10 min. Ubiquitinated proteins were immunoprecipitated with specific antibodies and analyzed by SDS-PAGE and Western blotting.

### Immunofluorescence assay

Cell samples were fixed with 4% paraformaldehyde for 30 minutes, permeabilized with 0.2% Triton X-100, and blocked with 5% bovine serum albumin for 30 min. Primary antibody incubation was performed at a dilution of 1:400 overnight at 4 °C, followed by secondary antibody treatment for 2 h under light-protected conditions. Nuclear counterstaining was conducted with DAPI for 5 min prior to fluorescence microscopic imaging.

### Apoptosis analysis by flow cytometry

Cells were collected, washed with PBS, and centrifuged to remove supernatant. They were then resuspended in 1X binding buffer at a concentration of 2 × 10⁶ cells/mL. A 100-μL aliquot of the cell suspension was transferred to flow cytometry tubes, and 5 μL of Annexin V conjugate was added, followed by a 15-min incubation at room temperature in the dark. After centrifugation and resuspension in 1X binding buffer, 5 μL of PI viability dye was added, and the mixture was incubated for an additional 15 min on ice in the dark. Samples were analyzed by flow cytometry within 4 hours to ensure accurate apoptosis detection.

### Cell proliferation assays and Colony formation assay

OS cell viability was assessed using the CCK-8 assay (Solarbio, CA1210) as per the manufacturer’s instructions. Cells were seeded at 1000 cells per well in 100 µL and incubated at different time points. After adding 10 µL of CCK-8 reagent, the plates were incubated at 37 °C for 1 h, and absorbance was measured at 450 nm.

Digest logarithmic-phase monolayer cells with 0.05% trypsin to form single-cell suspensions in 10% FBS medium. Seed cells at a density suitable for their proliferative capacity into 6-well plates and incubate at 37 °C, 5% CO_2_ for 2 weeks. Monitor regularly and terminate when visible colonies form. Wash cells twice with PBS, fix with 4% PFA for 15 min, and stain with crystal violet for 20 min. Rinse with water, air-dry, and analyze stained areas using ImageJ.

### Wound healing assay and Transwell assay

Approximately 5 × 10⁵ cells were seeded into each well of a 6-well plate. The following day, after the cells had adhered, straight lines were drawn in each well using a pipette tip aligned with a ruler. The cells were then washed three times with PBS to remove dislodged cells, and fresh culture medium was added. The plates were incubated at 37 °C in a 5% CO_2_ incubator. Images were captured under a microscope at 0 h and 48 h to document the scratch wound healing process.

Cell migration was assessed using a 24-well transwell plate with 8-μm polyethylene terephalate membrane filters (Corning, 3422). MG-63 or Saos-2 cells were seeded in the upper chamber at 1 × 10^4^ cells per well in serum-free DMEM, while the lower chamber contained DMEM with 10% FBS. Cells were incubated for 24 h at 37 °C with 5% CO_2_. Non-migratory cells were removed with cotton swabs, and filters were fixed with 4% PFA for 15 min and stained with 0.5% crystal violet for 20 min before imaging.

### Quantification of triglycerides, total cholesterol and free fatty acid

The Amplex Red Triglyceride Assay Kit (Beyotime, S0219S), Amplex Red Cholesterol and Cholesteryl Ester Assay Kit (Beyotime, S0211S), and Amplex Red Free Fatty Acid Assay Kit (Beyotime, S0215S) were employed for the quantitative analysis of triglycerides, total cholesterol, and free fatty acids in cells, respectively, following the manufacturer’s protocols.

### Green neutral lipid stain

OS cells cultured on coverslips were fixed with 4% paraformaldehyde for 15 min and stained with the Staining Solution (Beyotime, C2053S) for 20 min. Immunofluorescence signals were visualized using confocal microscopy.

### Subcutaneous tumor formation in nude mice

Animal experiments were approved by the Medical Ethics Committee of the Ethics Committee of Wuhan Polytechnic University (BME-2025-2-11) and adhered to institutional guidelines. Twenty-four 4–5-week-old BALB/c nude mice were randomly assigned to four experimental groups under a double-blind protocol for group allocation and tumor monitoring. Mice were subcutaneously injected with 1 × 10⁷ MG-63 OS cells in the mid-posterior axillary region. Mice were administered EGCG at a dose of 30 mg/kg via intraperitoneal (i.p.) injection every three days for a total of three doses, with the treatment period spanning 28 days. Body weight and tumor volume were measured every 3 days for 4 weeks. Tumor volume was determined using the formula (L×W²)/2, with *L* denoting length and *W* representing width. In adherence to ethical standards and to maintain data integrity, animals were euthanized humanely prior to tumors reaching the maximum allowable size of 2000 mm³.

### Immunohistochemical (IHC) staining

Tissue sections underwent baking, de-waxing, and hydration, followed by antigen retrieval in Tris-EDTA buffer (pH 9.0) via microwave heating. After cooling and three 5-min PBS washes, endogenous peroxidase activity was blocked with 3% H_2_O_2_/methanol. Sections were incubated with normal serum to block nonspecific binding, followed by overnight incubation with Ki67, primary Anti-RHBDL2, Anti-USP3, Anti-PPT1, Anti-CTSK (Proteintech, 11239-1-AP), Anti-OCN (Proteintech, 16157-1-AP), and Anti-Runx2 (Proteintech, 20700-1-AP) antibody (1:200, 4 °C). Post-washing, sections were treated with enzyme-conjugated polymer secondary antibody. After repeated PBS-Tween washes, DAB staining was performed, followed by hematoxylin counterstaining, differentiation, dehydration through graded alcohols, xylene clearing, and neutral gum mounting.

### RNA sequencing (RNA-seq)

RNA was extracted from MG-63 cells treated with either control or three distinct RHBDL2-targeted shRNAs. Subsequently, the RNA samples underwent library preparation for RNA-seq with the VAHTS mRNA-seq V2 Library Prep Kit for Illumina (NR601-01, Vazyme). RNA-seq was performed by LC-Bio Technologies Co., Ltd (Hangzhou, China).

### DIA quantitative proteomics analysis

In brief, MG-63 cells following RHBDL2 knockdown (cells transfected with shNC served as the control group) were lysed. Protein extraction was performed using 8 M urea lysis buffer supplemented with protease inhibitors, followed by centrifugation (14,100 × *g*, 20 min) and Bradford quantification, with aliquots stored at −80 °C. For digestion, 100 µg proteins were reduced with 200 mM DTT (37°C, 1 h), alkylated, and digested with trypsin (1:50 ratio, 37 °C overnight). Peptides were acidified with 0.1% FA, desalted via C18 columns (pre-conditioned with ACN/0.1% FA), and lyophilized after elution with 70% ACN. LC-MS/MS analysis utilized a Q Exactive HF-X Orbitrap coupled to an EASY nLC 1200 system. Peptides (500 ng) were separated on a 25 cm C18 column (60 °C) with a 80-min gradient (8-40% B; A: 0.1% FA, B: 80% ACN/0.1% FA). DIA acquisition included one MS1 scan (60k resolution) and 42 variable isolation windows (14–312 m/z) with stepped NCE. All runs employed dynamic exclusion (16 s) and standardized AGC targets (3E6 MS1, 5E4 MS2). DIA MS data from fractionated and single-shot samples were analyzed using Spectronaut (v15.7) to generate a hybrid spectral library. Protein identification/quantitation utilized the UniProt Homo sapiens database (2023-03-07). DIA quantitative proteomics analysis was performed by Nanjing Yiwei Jianhua Biotechnology Co., Ltd (Nanjing, China).

### Immunoprecipitation and mass spectrometry (IP-MS)

OS cells were lysed, and the extracted proteins were subjected to overnight incubation with the RHBDL2 primary antibody at 4 °C on a rotator. Protein A + G Agarose (Beyotime, P2055) were then added to the mixtures and rotated for an additional 4 h at 4 °C. Following centrifugation and washing, the beads were collected and boiled to elute the bound proteins. These proteins were subsequently analyzed by SDS-PAGE, followed by Western blotting or HPLC-MS/MS. HPLC-MS/MS analysis was conducted at Beijing Qinglian Baiao Biotechnology Co., Ltd (Beijing, China).

### Molecular docking analysis

The human RHBDL2 protein structure (UniProt ID: AF-Q9NX52-F1) was retrieved from the UniProt database. Binding pockets were predicted using CASTpFold, with the top-ranked pocket selected for high-throughput docking. Virtual screening was performed using the Dock Ligand (LibDock) module in Discovery Studio against three compound libraries: (1) Enamine FDA-Approved Drugs Collection (1040 compounds), (2) Enamine fda_p0.0 (3180 compounds), and (3) Targetmol Natural Compound Library (2960 compounds). Following docking score ranking, the top 1% of compounds (*n* = 72) underwent detailed evaluation, yielding five prioritized candidates: Imatinib, Luteolin 3′-O-β-D-glucuronide, Epigallocatechin gallate, Calceolarioside B, and Baicalin methyl ester. To validate binding stability, 100-ns molecular dynamics simulations were conducted in Schrödinger Desmond under the NPT ensemble (orthorhombic box, 300 K, 1.013 bar).

### Statistical analysis

All bioinformatical analyses were conducted using R software (version 4.4.2). Survival curves were generated using the Kaplan-Meier method and compared through log-rank tests. Overall survival (OS) was calculated from surgery to death date or last follow-up. Patients without events were censored at the last follow-up date. Statistical significance was set at *P* < 0.05.

All statistical analyses were conducted by means of GraphPad Prism 8.0, and graphs presented mean ± SD unless otherwise stated. The numbers of experiments are detailed in the Figure legends. The Wilcoxon test was employed for pairwise comparisons. Pearson analysis was applied for correlation analysis. Unpaired two-tailed Student’s *t*-tests were employed for pairwise comparisons, while one-way ANOVA along with Dunnett’s or Tukey’s post-hoc tests were utilized to evaluate differences among multiple conditions.

## Supplementary information

Original uncut gel figures

Supplementary Material 1

## Data Availability

Data is provided within the manuscript or supplementary information files. The mass spectrometry proteomics data have been deposited to the ProteomeXchange Consortium (https://proteomecentral.proteomexchange.org) via the iProX partner repository with the dataset identifier PXD062405. RNA-seq data have been deposited to the NCBI SRA database (https://dataview.ncbi.nlm.nih.gov/object/PRJNA1438394?reviewer=ceov66agn8da4fcijodpekuokj) with the identifier PRJNA1438394.
