# Supplementary figures and images for: RHBDL2 drives lipid metabolic reprogramming in osteosarcoma via USP3-mediated deubiquitination of PPT1
Source: Cell Death Dis. 2026 Apr 24;17(1):548. doi: 10.1038/s41419-026-08788-w (PMC13243514; doi:10.1038/s41419-026-08788-w)

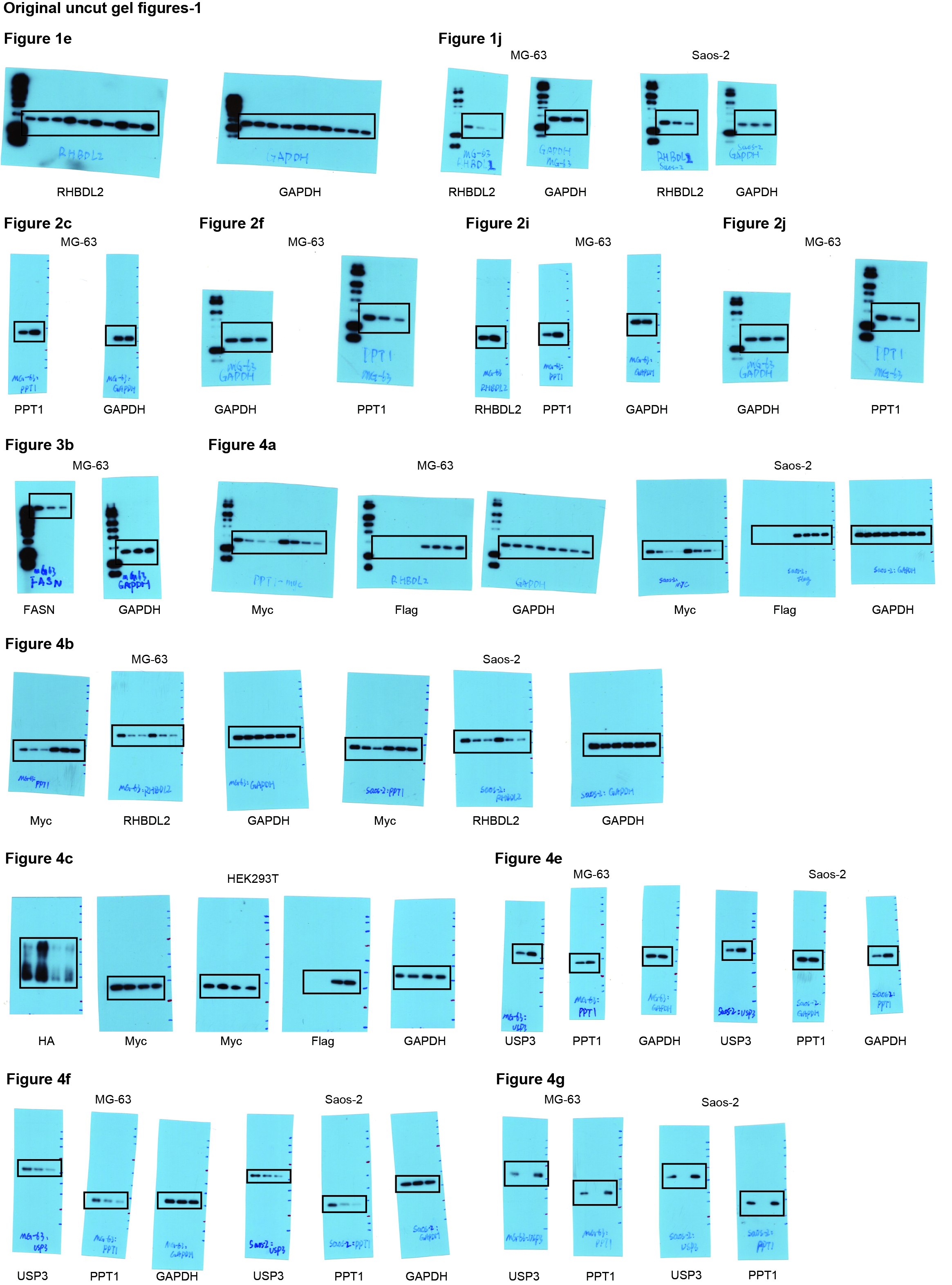

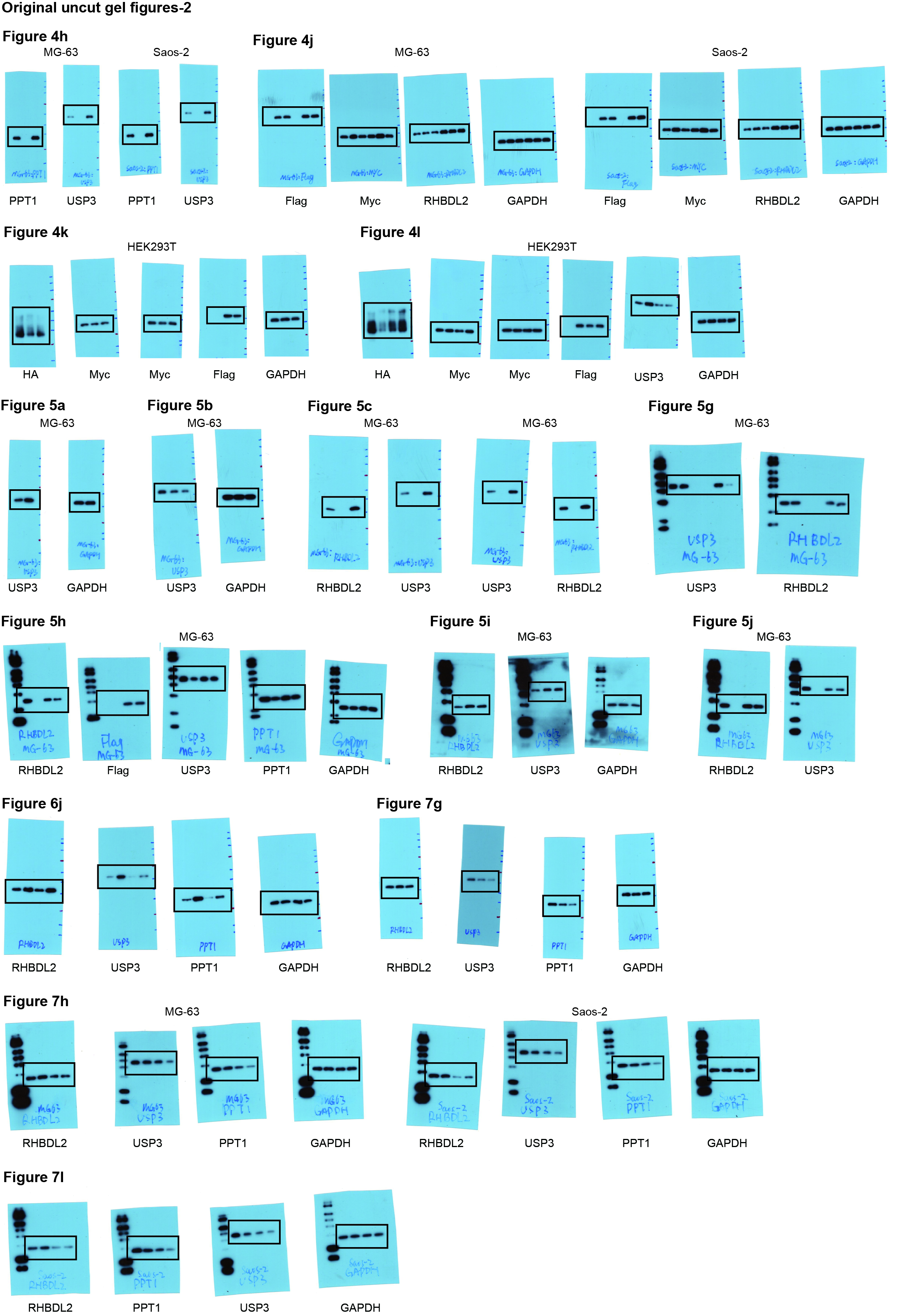

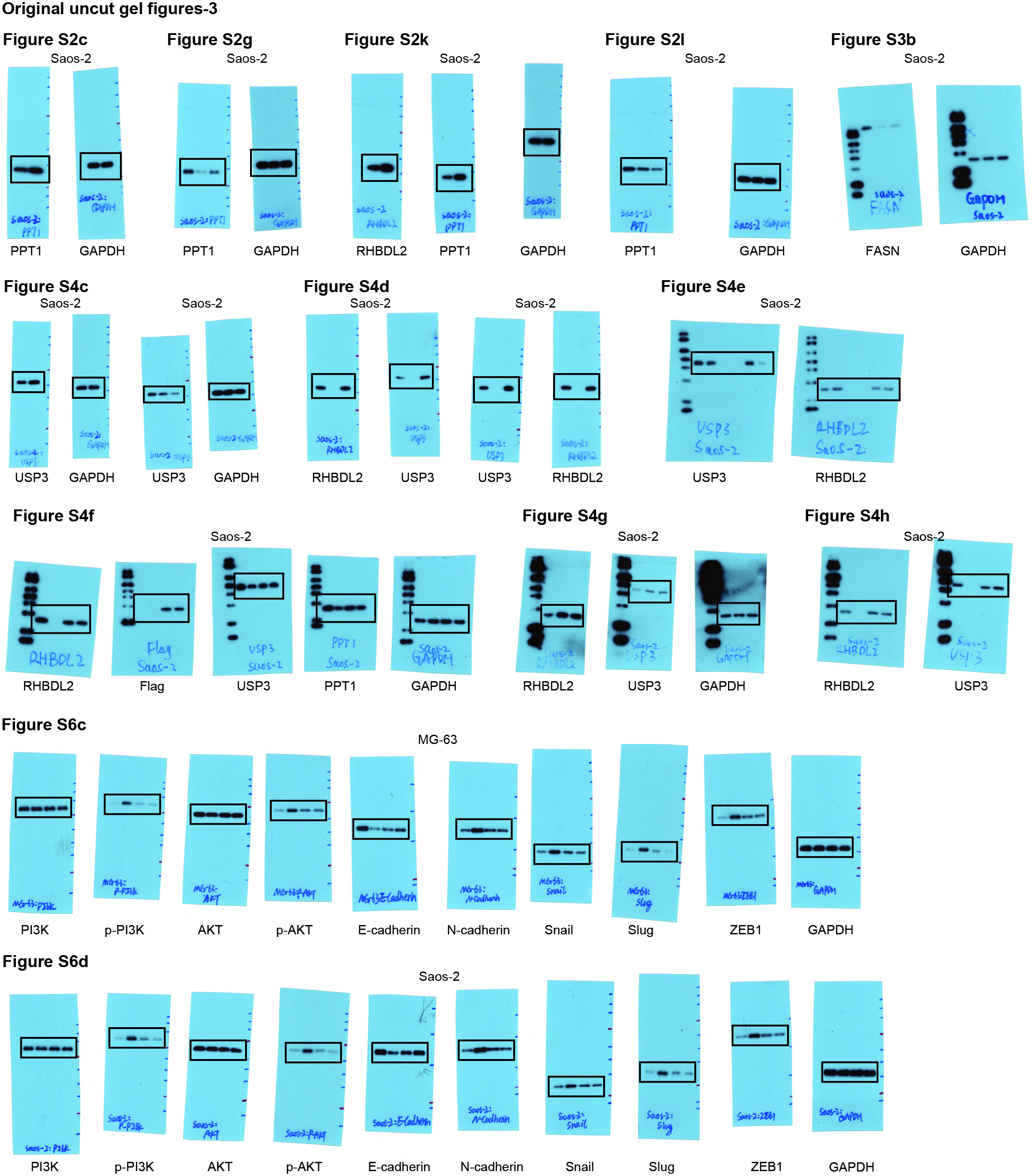

Supplement: Supplementary file 1 — Original uncut gel figures [file 41419_2026_8788_MOESM1_ESM.doc]
