# Supplementary material for: RHBDL2 drives lipid metabolic reprogramming in osteosarcoma via USP3-mediated deubiquitination of PPT1
Source: Cell Death Dis. 2026 Apr 24;17(1):548. doi: 10.1038/s41419-026-08788-w (PMC13243514; doi:10.1038/s41419-026-08788-w)
Supplement: Supplementary file 2 — Supplementary Material 1 [file 41419_2026_8788_MOESM2_ESM.docx]

**Supplementary File**

**
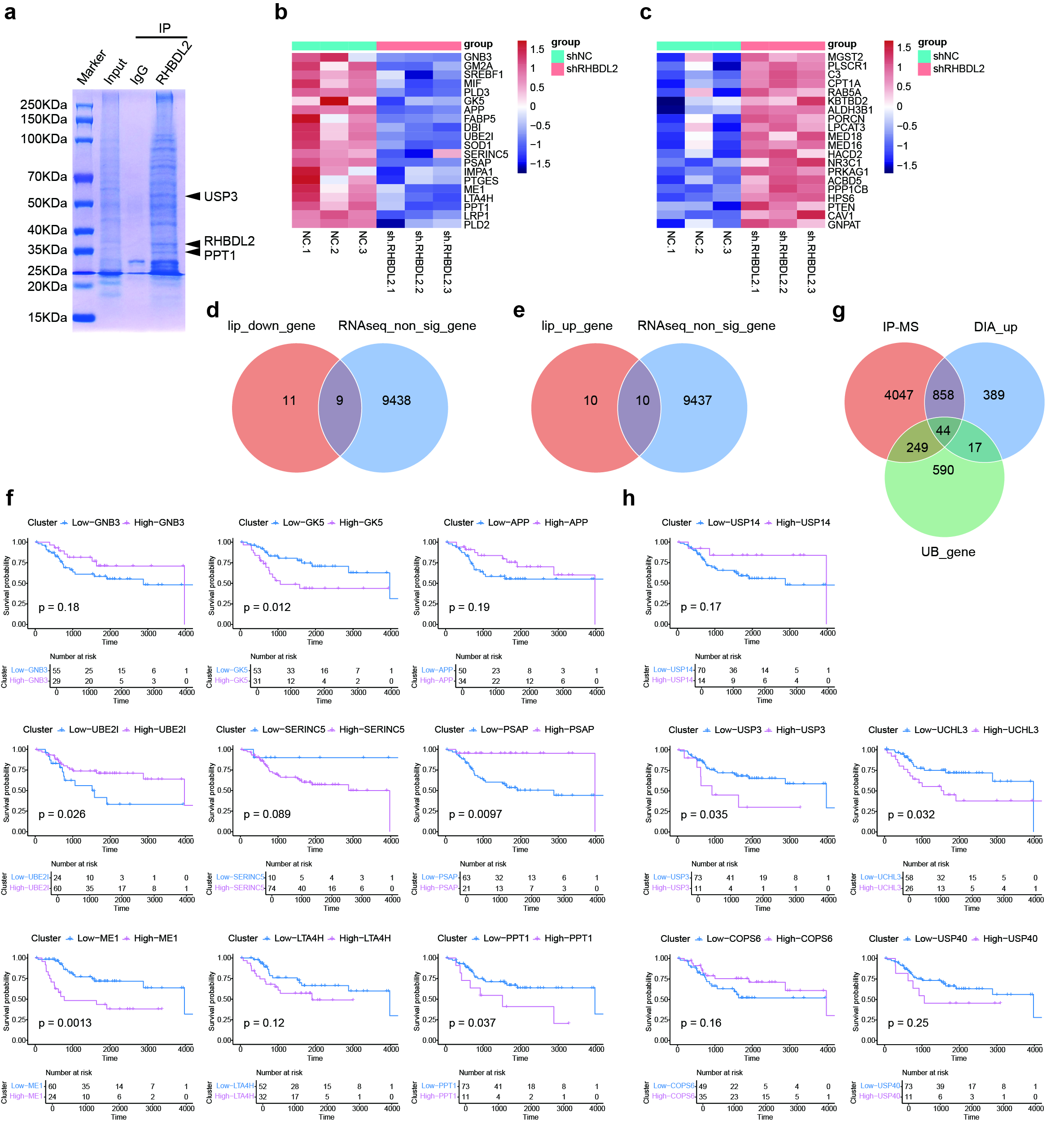
Supplementary Fig. 1 Purpose genes were screened using IP-MS, DIA, and RNA-seq. a** RHBDL2 was immunopurified by protein A/G beads in MG63 cells. The SDS-PAGE gel was stained with Coomassie Brilliant Blue. The protein bands were excised and digested in gel with trypsin followed by HPLC-MS/MS analysis. **b** Heatmap of the top20 downregulated lipid metabolism genes in the shRHBDL2 group compared to shNC. The color scale indicates the log2 fold change of gene expression. **c** Heatmap of the top20 upregulated lipid metabolism genes in the shRHBDL2 group compared to shNC. The color scale indicates the log2 fold change of gene expression. **d** Venn diagram illustrating the overlap between downregulated lipid metabolism genes in (**b**) and non-significant RNA-seq genes in the shRHBDL2 group compared to shNC. **e** Venn diagram illustrating the overlap between upregulated lipid metabolism genes in (**c**) and non-significant RNA-seq genes in the shRHBDL2 group compared to shNC. **f** Kaplan-Meier survival analysis of OS patients stratified by GNB3, GK5, APP, UBE2I, SERINC5, PSAP, ME1, LTA4H, and PPT1 expression levels (Low vs. High). **g** Venn diagram illustrating the overlap between IP-MS genes, upregulated DIA genes and UB_genes. **h** Kaplan-Meier survival analysis of OS patients stratified by USP14, UCHL3, COPS6, USP3, and USP40 expression levels (Low vs. High).


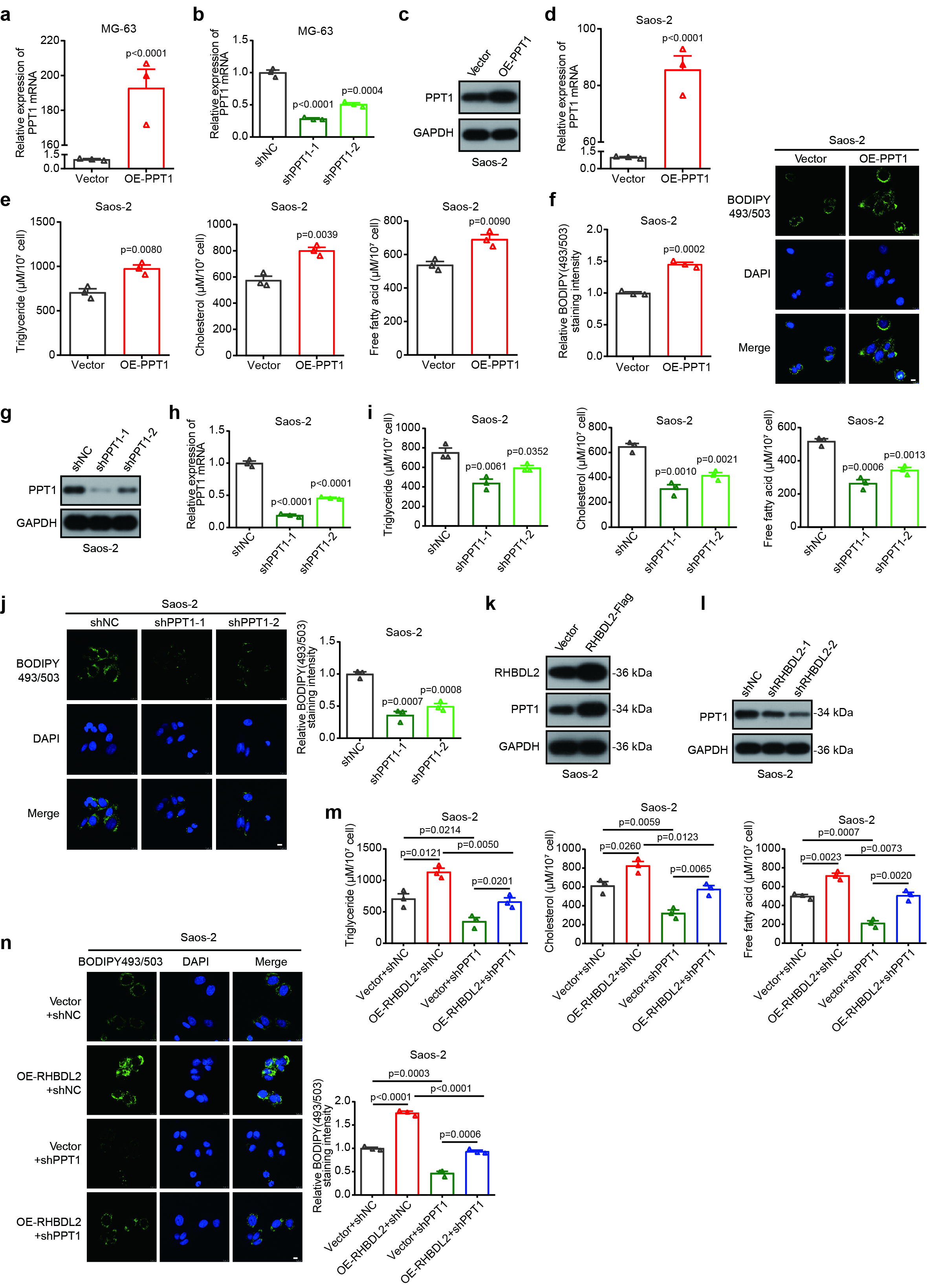


**Supplementary Fig. 2 RHBDL2 governs the lipid metabolism of OS cells through PPT1. a, b** Relative mRNA expression of PPT1 in MG-63 cells with PPT1 overexpression (**a**) or knockdown (**b**). **c** Western blot of PPT1 protein levels in Saos-2 cells with PPT1 overexpression. **d** Relative mRNA expression of PPT1 in Saos-2 cells transfected with vector or OE-PPT1. **e** Triglyceride, cholesterol, and free fatty acid levels in OE-PPT1 cells. **f** Lipid droplet accumulation (BODIPY493/503 staining, DAPI nuclei) and quantification in OE-PPT1 cells. Scale bar 10 μm. **g** Western blot analysis showing the protein expression levels of PPT1 in Saos-2 cells with PPT1 knockdown. **h** Relative mRNA expression of PPT1 in Saos-2 cells with PPT1 knockdown. **i** Triglyceride, cholesterol, and free fatty acid levels in PPT1-knockdown cells. **j** Lipid droplet imaging and quantification in PPT1-knockdown cells. Scale bar 10 μm. **k** Western blot analysis showing the expression of RHBDL2 and PPT1 in Saos-2 cells with RHBDL2 overexpression. **l** Western blot analysis showing the expression of PPT1 in Saos-2 cells with RHBDL2 knockdown. **m** Quantification of triglyceride, cholesterol, and free fatty acid levels in Saos-2 cells co-transfected with vector or OE-RHBDL2 and shNC or shPPT1. **n** Lipid droplet quantification and imaging in Saos-2 cells co-transfected with vector or OE-RHBDL2 and shNC or shPPT1. Scale bar 10 μm. Data shown in (a, b, d, e, f, h, i, j, m, n) as mean ± SD (n=3 biologically independent experiments); unpaired t-test for significance.


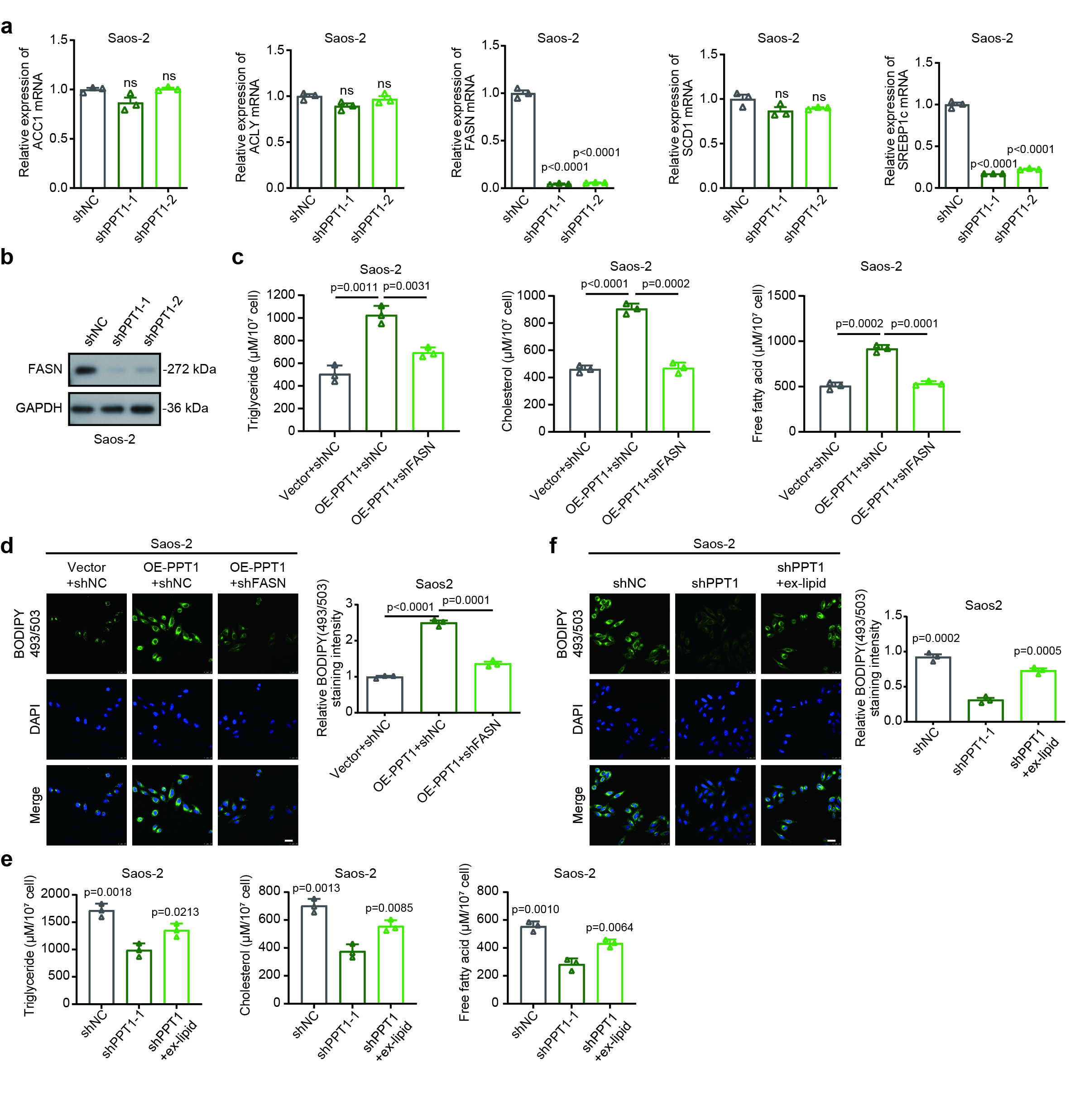


**Supplementary Fig. 3 PPT1 regulates FASN-dependent lipogenesis in Saos-2 osteosarcoma cells. a** Relative mRNA expression of ACC1, ACLY, FASN, SCD1, and SREBP1c in Saos-2 cells transfected with shNC, shPPT1-1, or shPPT1-2. **b** Western blot analysis of FASN protein levels in PPT1-knockdown Saos-2 cells. **c** Quantification of triglyceride, cholesterol, and free fatty acid levels in Saos-2 cells co-transfected with vector or OE-PPT1 and shFASN. Data shown as mean ± SD (n=3 biologically independent experiments); unpaired t-test for significance. **d** Lipid droplet analysis in Saos-2 cells co-transfected with vector or OE-PPT1 and shFASN. Scale bar 25 μm. Data shown as mean ± SD (n=3 biologically independent experiments); unpaired t-test for significance. **e-f** Exogenous lipid supplementation rescues the metabolic and functional deficits induced by PPT1 knockdown in Saos-2 cells. **e** Quantification of triglyceride, cholesterol, and free fatty acid levels. **f** Lipid droplet imaging and quantification. Scale bar 100 μm. Data shown as mean ± SD (n=3 biologically independent experiments); unpaired t-test for significance.

**
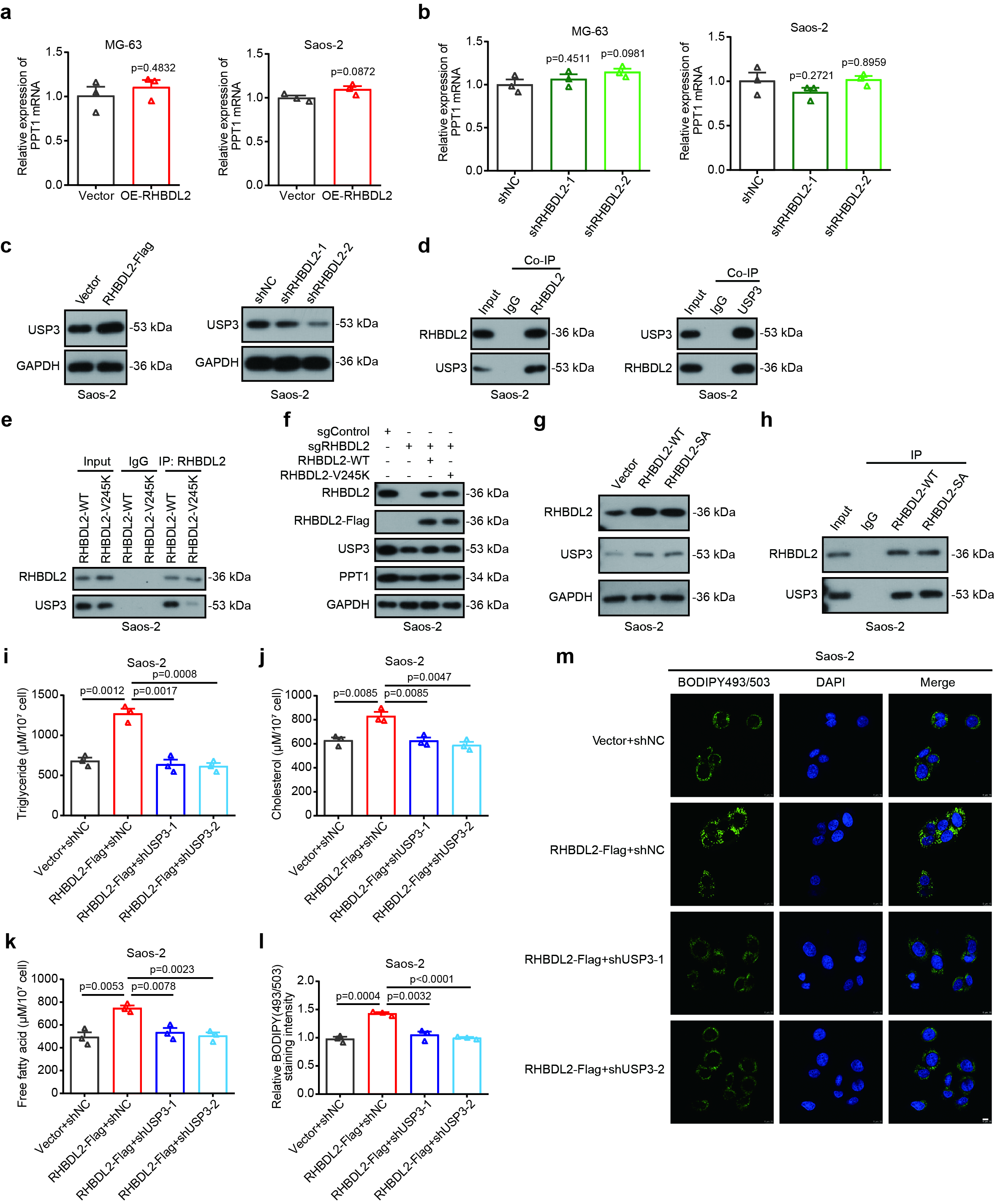
Supplementary Fig. 4 RHBDL2 requires USP3 to regulate lipid metabolism in Saos-2 cells. a** Relative mRNA expression of PPT1 in OS cells transfected with vector or OE-RHBDL2. Data shown as mean ± SD (n=3 biologically independent experiments); unpaired t-test for significance. **b** Relative mRNA expression of PPT1 in OS cells transfected with shNC, shRHBDL2-1, or shRHBDL2-2. Data shown as mean ± SD (n=3 biologically independent experiments); unpaired t-test for significance. **c** Western blot analysis shows USP3 expression in Saos-2 cells with RHBDL2 overexpression or knockdown. **d** Co-IP showing USP3 interaction with RHBDL2. **e** Co-IP assay showing the interaction between RHBDL2 and USP3 in Saos-2 cells. RHBDL2-V245K mutation significantly reduces binding to USP3 compared to RHBDL2-WT. **f** Western blot analysis of RHBDL2, Flag-tagged RHBDL2 variants, USP3, and PPT1 expression in Saos-2 cells across the indicated groups: sgControl, sgRHBDL2, sgRHBDL2 + RHBDL2-WT, and sgRHBDL2 + RHBDL2-V245K. **g** Western blot analysis of RHBDL2 and USP3 protein expression in cells transfected with a control vector, wild-type RHBDL2 (OE-RHBDL2-WT), or the RHBDL2-SA mutant (OE-RHBDL2-SA). **h** Co-IP demonstrating the interaction of USP3 with wild-type (WT) and SA-mutant RHBDL2, contrasted with Input and IgG controls. **i-k** Quantification of triglyceride (**i**), cholesterol (**j**), and free fatty acid levels (**k**) in Saos-2 cells co-transfected with vector or RHBDL2-Flag and shNC or shUSP3-1/-2. Data shown as mean ± SD (n=3 biologically independent experiments); unpaired t-test for significance. **l** Lipid droplet quantification in Saos-2 cells co-transfected with Vector or RHBDL2-Flag and shNC or shUSP3-1/-2. Data shown as mean ± SD (n=3 biologically independent experiments); unpaired t-test for significance. **m** Lipid droplet (BODIPY493/503 staining, DAPI nuclei) imaging in Saos-2 cells co-transfected with vector or RHBDL2-Flag and shNC or shUSP3-1/-2. Scale bar 10 μm.

**
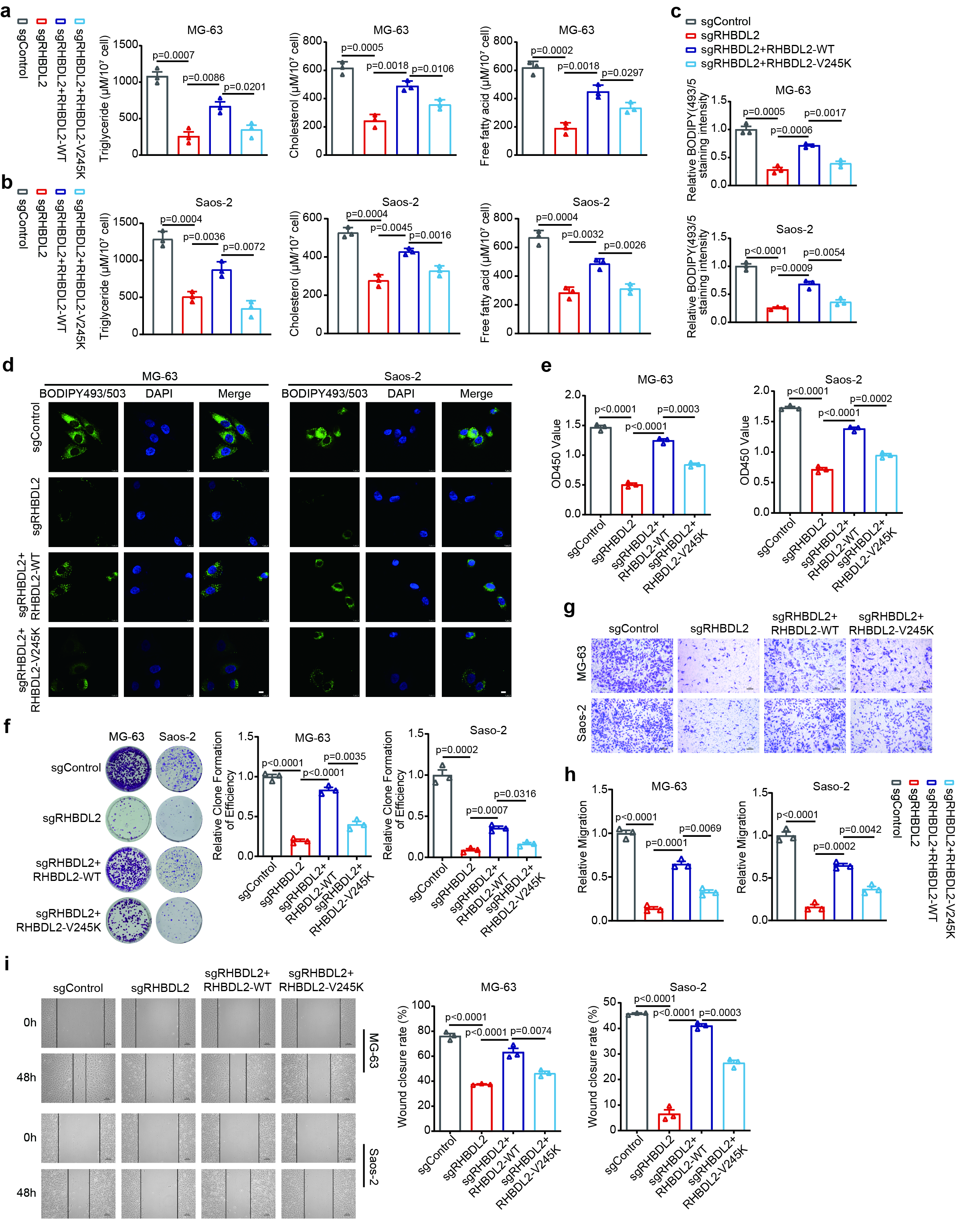
**

**Supplementary Fig. 5 The RHBDL2-V245K mutant impairs USP3 interaction, lipid metabolism, and oncogenic phenotypes in OS cells.**  **a, b** Quantification of triglyceride, cholesterol, and free fatty acid levels in MG-63 (**a**) and Saos-2 (**b**) cells under the specified conditions. **c, d** Analysis of neutral lipid content. Quantification of BODIPY 493/503 fluorescence intensity (**c**), and representative fluorescent images of BODIPY 493/503 (green) and DAPI (blue) staining (**d**) in MG-63 and Saos-2 cells. Scale bar 10 μm. **e** Cell viability measured by CCK-8 assay. **f** Colony formation ability assessed by crystal violet staining. Representative images (left) and quantitative analysis (right) are shown. **g, h** Cell migration evaluated by Transwell assay. Representative images (**g**) and quantitative analysis (**h**) are presented. Scale bar 100 μm. **i** Cell migration assessed by wound healing assay. Representative images at 0 and 48 hours and quantification of wound closure rate are shown. Scale bar 100 μm. Data shown in (a, b, c, e, f, h, i) as mean ± SD (n=3 biologically independent experiments); unpaired t-test for significance.


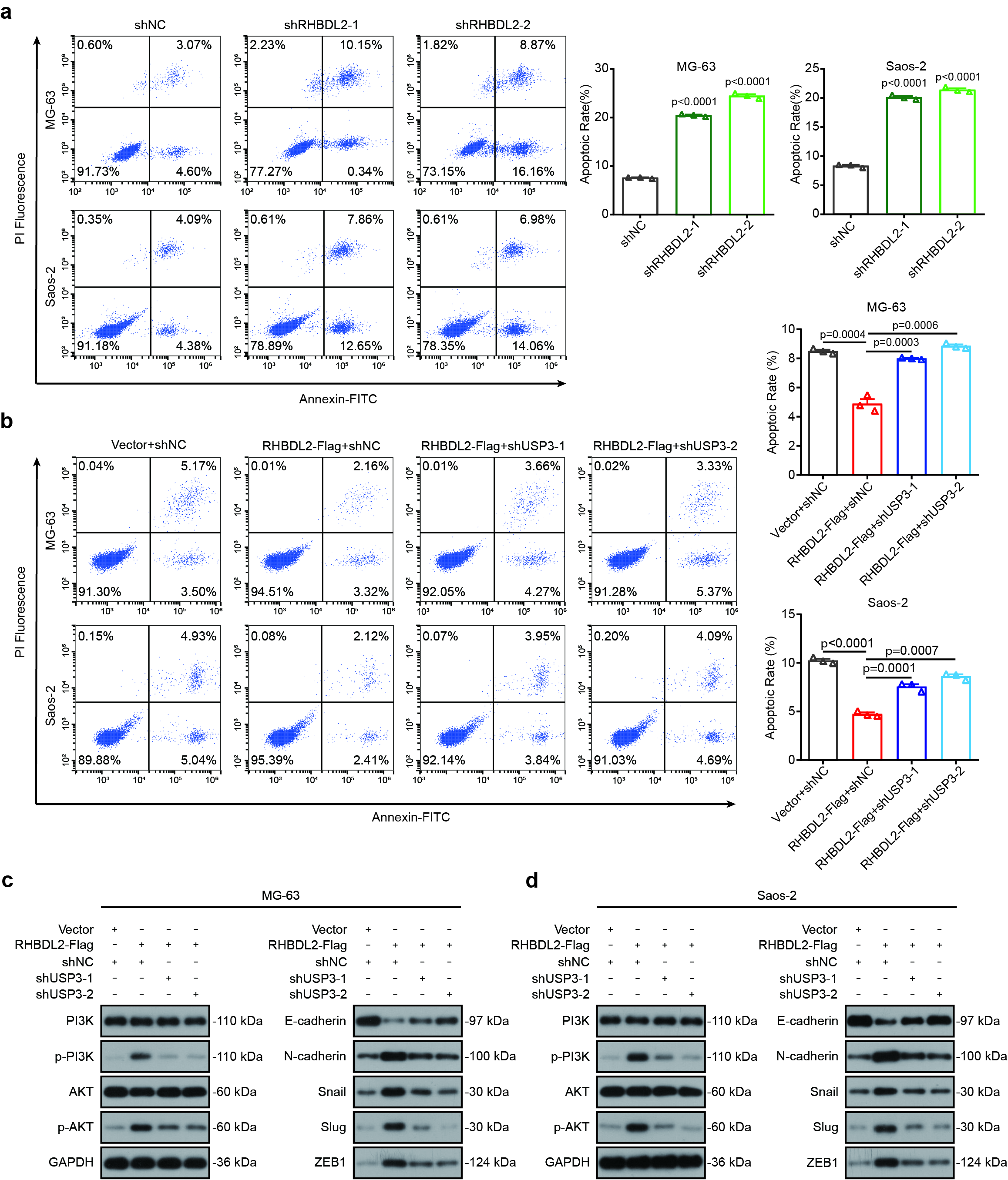


**Supplementary Fig. 6 RHBDL2 regulates apoptosis, PI3K-AKT signaling pathway and epithelial-mesenchymal transition (EMT) markers in OS cells. a** Flow cytometry analysis showing the apoptotic rates of MG-63 and Saos-2 cells transfected with shNC, shRHBDL2-1, or shRHBDL2-2. Data shown as mean ± SD (n=3 biologically independent experiments); unpaired t-test for significance. **b** Flow cytometry analysis demonstrating the apoptotic rates of MG-63 and Saos-2 cells transfected with Vector+shNC, RHBDL2-Flag+shNC, RHBDL2-Flag+shUSP3-1, or RHBDL2-Flag+shUSP3-2. Data shown as mean ± SD (n=3 biologically independent experiments); unpaired t-test for significance. **c, d** Western blot analyses of the PI3K-AKT signaling pathway downstream target genes (PI3K, p-PI3K, AKT, and p-AKT) and genes involved in EMT (E-cadherin, N-cadherin, Snail, Slug and ZEB1) in the MG-63 (**c**) and Saos-2 (**d**) cells.


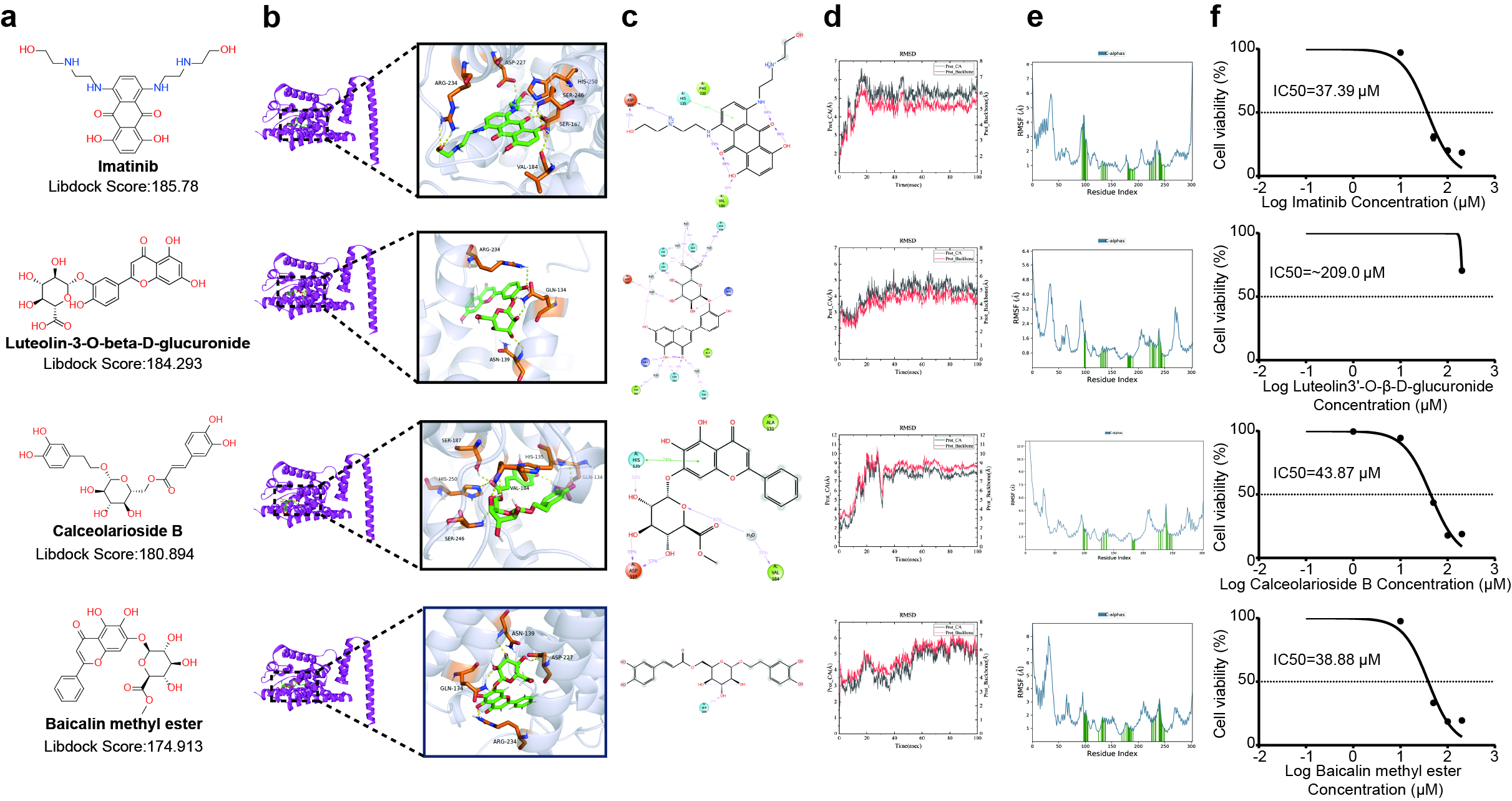


**Supplementary Fig. 7 Screening for small-molecule inhibitors targeting the interaction between RHBDL2 and USP3. a** The chemical structures and docking scores of Imatinib, Luteolin 3′-O-β-D-glucuronide, Calceolarioside B, and Baicalin methyl ester are presented. **b** Global views of the complexes formed by Imatinib, Luteolin 3′-O-β-D-glucuronide, Calceolarioside B, and Baicalin methyl ester are depicted. **c** Detailed views of the complexes formed by Imatinib, Luteolin 3′-O-β-D-glucuronide, Calceolarioside B, and Baicalin methyl ester are shown. **d, e** The dynamics of the complexes of Imatinib, Luteolin 3′-O-β-D-glucuronide, Calceolarioside B, and Baicalin methyl ester with RHBDL2 are illustrated by RMSD (**d**) and RMSF (**e**) plots. **f** CCK-8 assay was employed to evaluate the inhibitory effects of Imatinib, Luteolin 3′-O-β-D-glucuronide, Calceolarioside B, and Baicalin methyl ester on osteosarcoma cells.

Table S1 Clinicopathological characteristics of RHBDL2 expression in OS patients.

| **Character** | **RHBDL2-Low** | **RHBDL2-High** | **total** | **p value** | **r value** |
| --- | --- | --- | --- | --- | --- |
| **age** |  |  |  | 1 | 0.117 |
| <= 20 | 9 | 22 | 31 |  |  |
| > 20 | 12 | 29 | 41 |  |  |
| **sex** |  |  |  | 0.598 | 0.138 |
| Female | 9 | 18 | 27 |  |  |
| Male | 12 | 33 | 45 |  |  |
| **T** |  |  |  | 0.017 | 0.233 |
| T1 | 14 | 17 | 31 |  |  |
| T2_3 | 7 | 34 | 41 |  |  |
| **N** |  |  |  | 0.315 | 0 |
| N0 | 21 | 47 | 68 |  |  |
| N1 | 0 | 4 | 4 |  |  |
| **TNM** |  |  |  | 0.315 | 0 |
| TNM 1_2 | 21 | 47 | 68 |  |  |
| TNM 3_4 | 0 | 4 | 4 |  |  |

Table S2 List of partial proteins identified by Mass Spectrometry.

| **Accession** | **Gene Name** | **Peptides** | **Unique Peptides** | **MW [kDa]** | **Score Sequest HT: Sequest HT** |
| --- | --- | --- | --- | --- | --- |
| P16520 | GNB3 | 2 | 1 | 37.2 | 2.76 |
| Q6ZS86 | GK5 | 1 | 1 | 59.1 | 2.06 |
| P05067 | APP | 2 | 2 | 86.9 | 0 |
| P63279 | UBE2I | 4 | 4 | 18 | 14.66 |
| Q86VE9 | SERINC5 | 1 | 1 | 47 | 5.13 |
| P07602 | PSAP | 6 | 6 | 58.1 | 17.08 |
| P48163 | ME1 | 3 | 3 | 64.1 | 4.02 |
| P09960 | LTA4H | 20 | 20 | 69.2 | 76.87 |
| P50897 | PPT1 | 1 | 1 | 34.2 | 3.77 |
| P15374 | UCHL3 | 2 | 2 | 26.2 | 5.34 |
| P54578 | USP14 | 8 | 8 | 56 | 26.76 |
| Q9Y6I4 | USP3 | 1 | 1 | 58.9 | 2.42 |
| Q7L5N1 | COPS6 | 4 | 4 | 36.1 | 13.24 |
| Q9NVE5 | USP40 | 2 | 2 | 140 | 3.04 |

Table S3 Five candidate compounds interacting with RHBDL2.

| **Name** | **CAS** | **Libdock Score** | **Structure** |
| --- | --- | --- | --- |
| Imatinib | 70476-82-3 | 185.78 |  |
|  |  |  |  |
|  |  |  |  |
|  |  |  |  |
|  |  |  |  |
|  |  |  |  |
| Luteolin 3′-O-β-D-glucuronide | 53527-42-7 | 184.293 |  |
|  |  |  |  |
|  |  |  |  |
|  |  |  |  |
|  |  |  |  |
| Epigallocatechin gallate | 989-51-5 | 182.261 |  |
|  |  |  |  |
|  |  |  |  |
|  |  |  |  |
|  |  |  |  |
|  |  |  |  |
| Calceolarioside B | 105471-98-5 | 180.894 |  |
|  |  |  |  |
|  |  |  |  |
|  |  |  |  |
| Baicalin methyl ester | 82475-03-4 | 174.913 |  |
|  |  |  |  |
|  |  |  |  |
|  |  |  |  |
|  |  |  |  |
|  |  |  |  |
|  |  |  |  |
